# Incomplete penetrance in inborn errors of immunity: A skeleton in the closet—The sequel

**DOI:** 10.70962/jhi.20250064

**Published:** 2025-08-18

**Authors:** Dusan Bogunovic

**Affiliations:** 1 Department of Pediatrics, Department of Genetics, Center for Genetic Errors of Immunity, Columbia University Medical Center, New York, NY, USA

## Abstract

Primary immunodeficiencies (PIDs), more recently renamed inborn errors of immunity (IEIs), are a diverse group of over 550 genetic disorders. They cause clinically apparent immune dysregulation, leading to infections, autoinflammation, autoimmunity, and cancer. Initially, most IEIs were described as Mendelian disorders with complete penetrance, but the community has now shown that, in most IEIs, some individuals harboring disease-causing genotypes display only partial clinical disease, or no disease at all. Thus, most IEIs are actually Mendelian disorders with incomplete penetrance. Despite the frequency of incomplete penetrance in IEIs, the conceptual framework for systematically categorizing and explaining these occurrences remains limited. Here, I expand on four recurrent themes of incomplete penetrance that we have recently proposed: genetic variant quality, epigenetic and genetic modification, environment, and mosaicism. For each of these principles, I review what is known and unknown and propose future experimental approaches to fill the gaps in our knowledge. I focus on IEIs, but these concepts can be generalized to all genetic diseases.

## Teaser

Incomplete penetrance in genetic disorders can be influenced by genetic variant quality, genetic and epigenetic modification, environment, and mosaicism.

## Introduction

Primary immunodeficiencies (PIDs) or inborn errors of immunity (IEIs) are a heterogeneous group of monogenic lesions, resulting in severe infections, disorders of immune hyperactivation, or cancers. Since the first descriptions of inherited immunodeficiency in the 1930–1950s (1, 2, 3, 4, 5), IEIs have, by and large, been considered to be Mendelian disorders. In the 2010s, with the fall in costs for next-generation sequencing, the number of genetic errors identified as causing IEIs has grown exponentially, now exceeding 550 unique entities (6). These discoveries have often improved patient treatment (7, 8, 9) and have significantly advanced our understanding of basic and clinical immunology. However, despite unprecedented successes in this field, there is an “elephant in the room”: these disorders are widely held to be Mendelian, but they mostly display an imperfect segregation of gene variants with disease traits.

In genetics generally, the term incomplete penetrance is used to describe the absence of clinical disease in individuals harboring a known disease-causing genotype. This term makes it possible to get around the problem of our lack of precise understanding of incomplete penetrance for the moment, while allowing us to continue to describe genes as Mendelian, albeit with incomplete penetrance.

Before going into the details, we need to establish with precision the language and terminology used. Incomplete penetrance and reduced penetrance are considered here to be synonymous. As defined above, penetrance is the binary presence or absence of the disease trait in the presence of the causal genotype. However, a genetic defect may also present on a scale of disease severity or with different clinical phenotypes—a concept known as variable expressivity. Here, I will consider variable expressivity under the umbrella term incomplete penetrance, as the two phenomena often have largely overlapping origins. The terms fully penetrant and monogenic are not synonymous, as either may occur in the absence of the other. However, both these features are often considered necessary for a trait to be considered Mendelian.

Incomplete penetrance is common (10), but its exact incidence is difficult to determine from published studies. When considered, penetrance is typically assessed in the relatives of affected patients, with segregation of the disease traced from the proband. The reported rates of penetrance of specific IEIs, calculated in this manner, range from extremely low at about 5–10% (11, 12) to moderate 30% (13) and right up to almost 100% (14, 15). A recent study evaluating 453 patients from 193 families determined that the highest form of variable disease expressivity existed in familial lymphoproliferation, autoimmunity and malignancy STK4 deficiency, DNMT3B deficiency, and ATM deficiency, while immunological differences were prominent in syndromic and non-syndromic combined immunodeficiencies (10). Composite estimates across all IEIs indicate that ∼9% families display some degree of incomplete penetrance (16), although this frequency is probably closer to 20–30% across IEIs, given likely studies’ limitations. We suggest that there are at least two inherent biases underlying a pronounced underreporting of incomplete penetrance. These biases are: (1) reporting bias, due to a failure to pursue the study of new variants with highly reduced penetrance or to publish the results of such studies or a failure to pursue the study of already reported variants or to publish the results for such variants, as they are already considered to be Mendelian with full penetrance, thereby reducing the impact of such studies and creating a disincentive for authors, and (2) ascertainment bias, due to an inability to detect asymptomatic individuals carrying variants in the general population alongside the bias of healthy volunteers representing general population in databases.

In population-scale genetic studies generally, beyond the domain of IEIs, it has been noted that an average individual possesses ∼200 rare variants (17), 50 of which have been reported to drive disease, and yet these individuals remain healthy (18). Similarly, one study pointed out that about 1 in 4,000 adults caries a variant for a severe Mendelian condition but remains healthy (19). These studies suggest that the phenomenon of incomplete penetrance is both widespread and underappreciated.

Due to these biases, more remains unknown than known about incomplete penetrance across genetics (20, 21, 22, 23, 24). However, the study of IEIs is blossoming, leading to the reporting of ever increasing numbers of cases of incomplete penetrance and the continual emergence of new patterns. Here, I will review incomplete penetrance across IEIs and wider genetic fields and continue to develop the four principles of incomplete penetrance we proposed 5 years ago (25), to continue the development of a conceptual framework of incomplete penetrance in IEIs and beyond. For each principle, as before, I will document what is known and then what is unknown, proposing testable hypotheses that could be used to advance our current understanding. This evidence, these principles, and new ideas will help to establish a blueprint for future studies focusing specifically on incomplete penetrance. These concepts can readily be generalized to genetic diseases generally, despite our focus here on IEIs.

## Principle I: Genetic variant quality

### What is known

The quality of the genetic variant, a term which I use as synonymous to variant severity, can have different effects on its biochemical, cellular, and clinical penetrance. We, therefore, need to define these different types of penetrance. Biochemical penetrance refers to assessments of genetic variation in a test tube rather than in vivo, in an isogenic system with a readout. A complete absence of the protein typically results in more serious biological defects than hypomorphic variants, although there are exceptions (7). Such biochemical assays may suggest functional deficits, but such deficits may not be observed in the patient’s cells. Cellular penetrance refers to assessments of the biological pathways or processes in which the gene product with a variant is involved, in cells isolated from the patients themselves, often measured when cells are studied in isolation with a specific assay (e.g., the signal transduction pathway of a mutated receptor) in a non-isogenic system. The cellular phenotype mostly depends directly on the severity of the genetic defect and is usually strongly correlated with biochemical results. Furthermore, clinical penetrance mostly tracks biochemical and cellular penetrance, although this is not always the case. We can, therefore, propose a simple model: the severity of the genetic defect determines biochemical dysfunction, which governs the degree of perturbation in immune cells and, therefore, the propensity for clinical manifestations.

This model holds for a few genetic disorders. One of the principal examples is IFNGR1 deficiency, the first specific genetic etiology of Mendelian susceptibility to mycobacterial disease due to either environmental mycobacteria (EM) or bacille Calmette–Guerin (BCG) immunization (26, 27) to be described. Complete deficiency due to autosomal recessive (AR) IFNGR1 defects invariably results in BCG or EM infections by the age of 5 years, and clinical penetrance is complete (28, 29). By contrast, subjects with partial IFNGR1 deficiency, which is typically autosomal dominant (AD), often remain asymptomatic for longer periods of time, have milder disease, or may, in some cases, never develop disease (30, 31, 32). By inference, with subsequent experimental demonstration, these AD forms are characterized by the retention of some activity when IFN-γ signaling is assayed in patient cells in vitro (30, 31). Some activity is better than none, and the retention of higher levels of activity is associated with a lower observed penetrance.

A similar situation has been observed for STAT1 loss-of-function (LOF) defects. AR complete deficiency with a complete absence of type I–III interferon signaling leads to the complete penetrance of lethal intracellular bacterial and viral infections (33, 34, 35). By contrast, AR partial deficiency, with low levels of signaling, leads to penetrant but milder intracellular bacterial disease (36, 37, 38), and AD forms with LOF point variants retaining some function cause predominantly mycobacterial disease, but with incomplete penetrance (33, 34, 39, 40, 41). These findings demonstrate that incomplete penetrance can be potentiated by partial deficiencies of essential genes but also by complete deficiencies of nonessential genes of type I–III IFN signaling, leaving some signaling still intact or allowing another related pathway to keep some common transcriptional programs active. This situation can readily be observed in deficiencies of *STAT2*, *TYK2*, *IFNAR1*, and *IFNAR2*, in which severe viral disease affects some but not all individuals, with a greater penetrance observed for infections with live-attenuated viral vaccines than for common viral infections of childhood (42, 43, 44, 45, 46, 47, 48, 49, 50, 51, 52).

Allele-penetrance associations have also been noted in other IEIs with infectious phenotype, like CARD9 deficiency (53, 54), and in other IEIs less clearly associated with infection, including variants of *STAT3* (55), *PRF1* (55, 56, 57, 58), and *AIRE* (59, 60). The functional impact of each variation should be studied in isolation but, at times, genetics alone can lead the way. For example, a study of a cohort of patients with congenital asplenia due to *RPSA* variants revealed marked incomplete penetrance, but with no predicted functional differences between incompletely and fully penetrant variants. However, all the missense variants with incomplete penetrance were located close together in the structure, as were those with complete penetrance. Similarly a structural defect of the noncoding *RPSA* mRNA resulted in incomplete penetrance, whereas a noncoding variant resulting in complete transcript decay conferred complete penetrance (61). These findings suggest that milder hypomorphic variants probably retain some residual function, which may be sufficient for normal spleen development in some individuals. Thus, even in the absence of a full understanding of the effect of variants, the severity of the defect can be seen to be associated with its penetrance.

Autoimmune lymphoproliferative syndrome (ALPS) initially appeared to fit this mold, in that penetrance seemed to be a function of the location of the *FAS* variant, the most common cause of ALPS. Homozygous or compound heterozygous forms of ALPS-FAS are fully penetrant and particularly severe, with an early onset and an often lethal outcome (62, 63, 64, 65). Heterozygous forms are less penetrant. There is also an additional hierarchy among AD variants: missense variants of the intracellular domain are more highly penetrant (63–90%) than those in the extracellular domain (30–52%). The dominant-negative (DN) mechanism of intracellular domain variants therefore leads to more severe apoptosis than the haploinsufficiency mechanism of extracellular domain variants (66). There is clearly a relationship between the nature of the variant and the probability of disease. However, some level of defective Fas-mediated apoptosis can be identified in almost all affected individuals, for all variants. Finally, some “asymptomatic” individuals even display lymphocyte expansions or have autoantibodies without clinical autoimmunity or true lymphoproliferation (67, 68, 69). One recent study evaluated over 165 cases of ALPS and not only identified associations between the domain in which the variant occurred and penetrance but also suggested additional mechanisms contributing to this extensive clinical variability, particularly in cases with no correlation between genotype and phenotype (70). Be that as it may, each variant requires careful assessment, and determination of the threshold beyond which subclinical cellular defects become clinically apparent disease is vital for our understanding of this model and to expand the spectrum of molecular events, which are rarely mutually exclusive.

### What remains unknown and future avenues for research

Despite these examples, the association between the degree of pathogenicity of a variant and its penetrance has not been clearly documented. Indeed, this model does not hold if a genetic defect is complete (deletions, frameshifts, etc.) but has variable penetrance. The best studied example of this is provided by CTLA4 haploinsufficiency. Intensive studies in large cohorts have reported no association between genotype and penetrance (71, 72, 73). For example, in a recent analysis, only 90 of 133 subjects from 54 unrelated families carrying 45 different *CTLA4* variants in the heterozygous state presented features of disease. The missense, nonsense, or frameshift nature of the pathogenic variant had no apparent bearing on penetrance. Furthermore, immunologic phenotyping and in vitro CTLA4 dysfunction results were similar for both affected and unaffected carriers of the variants, suggesting complete cellular penetrance (73). However, cellular penetrance is fully dependent on the phenotype in question and the sensitivity of the assay used. Thus, on closer examination, the loss of surface CTLA4 expression was found to be less severe in unaffected carriers (73). The degree of CTLA4 perturbation in cells is, therefore, correlated with disease presentation even though the CTLA4 genotype cannot explain disease segregation. Along similar lines, a more recent study suggested that LOF variants of *CLEC7A* can act as gene modifiers, accounting for penetrance in some cases (74). Interestingly, a related defect of T cell regulation constituting a more severe phenocopy of CTLA4 haploinsufficiency (LRBA deficiency, IPEX) has been shown to have almost complete penetrance (75, 76). There are, therefore, probably other disease modifiers, in addition to *CLEC7A* variants, that can affect the degree of T reg cell dysfunction.

Recent work also highlighted that individuals with *SPI1* (encodes PU.1) pathogenic variants that lead to haploinsufficiency also have highly reduced penetrance (77). Why this is the case is still not known. Also very interestingly, recent work highlighted, at least in part, why sexual dimorphisms exists in patients with variants in *NFKB1* causing common variable immunodeficiency (CVID). Notably, authors conclude that autoimmunity in NFKB1 haploinsufficiency females is secondary to defective XIST-dependent X chromosome inactivation in T cells (78). Why this happens remains to be molecularly and biochemically documented.

These and other cases (79, 80, 81) demonstrate that the most severe genetic defects are not always associated with the greatest propensity for disease. Alternative hypotheses are, therefore, required. Complete TBK1 deficiency was initially thought to result in more severe disease than DN variants. However, it was shown experimentally that DN variants resulted in a more profound defect of IFN-I induction as, unlike the complete absence of TBK1 protein, they prevented the recruitment of IKKe, which could partly rescue the phenotype (7). Indeed, as shown in previous studies, the most severe genetic variants may lead to more robust compensatory responses (82, 83). Transcriptional adaptation—the process by which frameshifts/nonsense variants activate the transcription of homologous genes—may rescue these complete deficiencies (82, 83). In such cases, whether due to experimental knockout or disease variants, nonsense-mediated decay triggers an upregulation of genes with a similar sequence predicted to have a partially overlapping function (82, 83). This phenomenon suggests an enticing and testable hypothesis to account for incomplete penetrance in asymptomatic carriers of disease-causing nonsense variants. The ability of this “genomic compensation” to rescue disease phenotypes should be a key focus of future studies.

## Principle II: Epigenetic and genetic modifiers can affect the penetrance of a variant

### What is known

Despite the very sparse experimental evidence, the mechanisms most commonly proposed to account for incomplete penetrance are epigenetic regulation and/or potential modifier genes. As next-generation sequencing (84) becomes more commonplace and epigenetic techniques are introduced into the study of IEIs, evidence is finally being obtained to substantiate these reasonable presumptions.

We have proposed the concept of autosomal random monoallelic expression (aRMAE) (25, 85). Unlike imprinting, in which one allele—the maternal or paternal allele—is completely silenced throughout the organism, aRMAE results from a somatic but mitotically stable commitment to biased expression in favor of one allele rather than the other. De facto, results in transcriptional mosaicisms, as at DNA level, each cell is heterozygous for the variant concerned, but some cells (a lineage or sublineage) are committed to biased expression of one allele rather than the other, whereas other cells or lineages continue to express both alleles. This transcriptional bias may result in different proportions (e.g., 99%, 80%, or 60%) of the transcripts obtained originating from the paternal or maternal allele, suggesting that the system displays plasticity. We have, thus, proposed the term “transcriptotype” for this situation, which may differ from predictions based on genotype. In one family with a *JAK1* gain-of-function variant, we documented suppression of the mutated allele in one healthy relative carrying the variant, across all cell types, whereas the proband had biallelic expression in T cells (85). Similarly a complete suppression of the mutated allele was documented in a family carrying a DN *STAT1* variant (85). The healthy father was heterozygous for the dominant allele at DNA level, but his transcriptotype revealed the presence of the WT mRNA only in all cell types tested, contrasting with his sick child, who was heterozygous for the dominant allele at DNA level and had similar levels of both transcripts in a monocyte subset (but with a suppression of the mutated allele in T cells). H3K27 methylation and DNA methylation were suggested as possible mechanisms governing these processes (85). Screening in healthy individuals suggested that 4% of all IEI genes can display aRMAE in healthy donors. However, it should be stressed that disease-causing variant alleles may confer a homeostatic advantage or disadvantage on the host, and as such, it is likely that significantly more genes causing IEIs display aRMAE when mutated, resulting in discordant genotypes and transcriptotypes.

CVID—the most common form of immune deficiency—is an ideal model for studies of incomplete penetrance. A report on CVID-discordant monozygotic twins suggested that the twin with CVID displayed higher levels of DNA methylation in critical B-cell genes (*PIK3CD*, *BCL2L1*, *RPS6KB2*, transcription factor 3 [*TCF3*], and *KCNN4*) (86). Similarly, a follow-up analysis of 23 CVID patients revealed defective demethylation of selected CpG sites during the transition from naive to switched memory B cells (87). More recently a single-cell epigenomics and transcriptomics census of naïve-to-memory B cell differentiation in CVID-discordant monozygotic twins also suggested a role for epigenetic signatures, such as DNA methylation and chromatic accessibility (88). Additional concrete and diagnostic evidence is largely lacking for CVID, but the hypothesis of a role for epigenetic markers is particularly attractive given the variable disease expressivity in CVID.

Epigenetics and the genetic control of epigenetics may, therefore, play a particularly important role in penetrance.

#### Modifier genes

COPA syndrome is an IEI caused by variants of the *COPA* gene. It displays AD inheritance with incomplete penetrance. COPA patients present with interstitial lung disease and pulmonary hemorrhage, with the subsequent development of arthritis (89). Interestingly, there is a considerable clinical overlap between STING-associated vasculopathy with onset in infancy and COPA syndrome in terms of lung inflammation (90). In situations such as this, the genetic and biochemical interactions should perhaps be examined closely if they are not already obvious, as they may have functional consequences leading to different disease outcomes. Indeed, one common *STING* allele has been shown to prevent clinical penetrance for the rare COPA syndrome. Carriers of the deleterious *COPA* allele were not affected by the disease if they also carried a fairly common *STING* allele, which silenced the biochemical activity of the deleterious COPA, neatly explaining the observed penetrance (91).

Monogenic variants causing CVID continue to be identified but account for only a fraction of cases. Not infrequently, in inherited CVID (∼10–20%), a polygenic etiology is suggested (92). Specifically, deleterious variants of *TNFRSF13B* (TACI) are present in 1% of healthy individuals in public databases but in 10% of those with CVID, suggesting that this genetic background contributes to CVID but cannot drive the CVID phenotype alone (93). An enrichment has been observed in variants of other genes, such as *TNFRSF13B*, *MSH5* and *BAFFR*, in cohorts of CVID patients, but these variants are also present in healthy populations and are, therefore, not sufficient to drive disease on their own (94, 95).

Digenic and, ultimately, polygenic inheritance can help us to analyze variable disease expressivity and incomplete penetrance. The idea is that a variant in cis or in trans disrupts biochemical epistasis, tipping the system toward disease. One recent study by Nomani et al. (96) did not address the issue of penetrance directly but suggested that disease inheritance is digenic in 66% of patients with adult-onset systemic autoinflammatory diseases. The combinations of genes involved in this digenic inheritance were *NOD2/MEFV*, *NOD2/NLRP12*, *NOD2/NLRP3*, and *NOD2/TNFRSF1A*. This discovery paves the way for detailed penetrance analyses in the context of both digenic and polygenic contributions to disease manifestations in families with these genetic variants. In another study, Massaad et al. reported that homozygosity for *NEIL3* variants caused a uniformly fatal immune disease (recurrent infections and severe autoimmunity) in one family, but clinically silent immune dysfunction in an unrelated healthy individual. As an explanation for this incomplete penetrance, they cited the presence of a cryptic duplicated homozygous variant of *LRBA*—defects of which are known to cause systemic autoimmunity, recurrent infections, and hypogammaglobulinemia—exclusively in the affected family. They tested this “double-hit hypothesis;” they generated *Neil3*-deficient mice, which, like their human counterparts, displayed no overt signs of autoimmunity until faced with a second environmental challenge, suggesting that environmental effects can potentiate a genotype. Disruption of the genetic epistasis between *NEIL3 *and *LRBA* remains a very attractive hypothetical mechanism, contributing to differences in disease penetrance (97).

Elegant experimental evidence for epistasis in CVID was obtained with the discovery of a de novo* TCF3* variant in a family already carrying a variant of the CVID-associated *TNFRSF13B* gene. The sick individual with both variants presented a severe CVID-like disorder and systemic lupus erythematosus. Family members with the *TNFRSF13B* variant only were asymptomatic or displayed only mild disease, and the son of the proband, who carried only the *TCF3* variant, displayed a partial clinical phenotype (98). The effect of having two variants, disrupting epistasis, was documented by clinical scoring for disease severity and by in vitro studies documenting the biological phenotype. The effects of these genes converged on immunoglobulin class-switching pathways, resulting in severe disease.

Similar epistatic regulation was documented in ALPS patients with variants of both *FAS* and *PRF1* (99) or *FAS* and *CASP10* (100), patients with hyperimmunoglobulinemia D and periodic fever syndrome with *MVK* and *TNFRSF1A* variants (101), patients with broad susceptibility to infections associated with *IFNAR1* and *IFNGR2* variants (102), patients with X-linked immunodeficiency caused by *XIAP* variant and a *CD40LG* polymorphism (103), and pediatric patients with inflammatory bowel disease, in which a known *NOD2* variant probably interacts with variants of GSDMB, ERAP2, or SEC16A (104).

In most of these cases, one of the two hits had previously been reported as the causal variant in isolation. This raises an obvious question, because if synergistic interactions between two or more genetic loci are required, how can one mutated locus be responsible? It is possible that these isolated cases due to *TCF3* (105) or *LRBA* alone (75, 76, 106) corresponded to milder forms of disease or that these “isolated” cases actually involved a second, unknown gene. Alternatively, these combinatorial genetic defects may result in blended phenotypes due to overlapping clinical disease resulting from the co-occurrence of two independent monogenic defects. Recently unusual features of Williams–Beuren syndrome (WBS), including recurrent infections and skin abscesses in a child, were shown to be due to heterozygosity for a 0.53-Mb deletion on chromosome 7q11.23, corresponding to the known cause of WBS, together with a biallelic loss of NCF1, leading to AR chronic granulomatous disease (107). Blended phenotypes are common in clinical genetics (∼5% of rare disease diagnoses) (108, 109) and can affect IEIs (107, 110, 111). Whether through blended phenotypes or epistasis, digenic inheritance is increasingly recognized as a determinant of expressivity and penetrance.

## What remains unknown and future avenues for research

Over the 5 years that have elapsed since our initial review, the numbers of epigenetic and combinatorial genetic hits, each probably surprising rare, have increased substantially. What remains largely unknown is the frequency with which common variants affect the incomplete penetrance of rare diseases. It also remains largely unknown how common the epigenetic control of WT vs. mutated allele transcription is in the incomplete penetrance of rare diseases. In epistasis, the modifier gene could plausibly be a common variant with a purely protective (or pathogenic) role acting in combination with a rare variant. This has already been shown for COPA, as described above, but is probably also the case in a few other monogenic forms of autoimmunity (e.g., APS1, IPEX, and CTLA4), in which relatively common autoimmunity-associated *HLA* alleles probably modify the risk of developing autoimmunity to specific autoantigens (59). It has also been suggested that X-linked variable immunodeficiency segregates with relatively common variants of *CD40LG* (103) and that susceptibility to familial Mediterranean fever is modified by interactions of *MEFV* variants with polymorphisms of *SAA1* (112). Inspired by large studies in other genetic disciplines in which such occurrences are well documented, focusing on non-syndromic midline craniosynostosis caused by rare *SMAD6* variants, for example, we should organize large registries of detailed, well-curated phenotypes (113). This would make it possible to document and, more importantly, to act on more subtle clinical signs and symptoms that are probably often missed. Studies of aggregate mutational burden in IEIs, in which the composite effects of many minor deleterious variants regulate disease risk, may indeed be as revealing as such studies have been for other types of rare diseases (114, 115, 116). Databases, such as UK Biobank, All of US, and BioMe, should be leveraged in addition to IEI registries, as the combination of these resources can tell us much about the degree to which clinical phenotypes are determined by particular genetic variants, their combined effects, and the situations in which protective alleles may have a particularly strong effect.

Clinical phenotypes form a spectrum with no clear distinguishing line between rare and common phenotypes, and the same is true for genetics. For example, rare variants leading to complete TYK2 deficiency result in monogenic susceptibility to Mycobacterium tuberculosis (TB) and Mendelian suspetibility to mycobacterial disease (MSMD) with relatively high penetrance (∼80%) (117, 118). Conversely, it was recently demonstrated that a common *TYK2* variant (allele frequency of 4.2% in Europeans) confers a predisposition to TB ( odds ratio [OR] 89.3) and MSMD (OR 23.5) in homozygous individuals living in endemic regions. The estimated penetrance was ∼80% for TB and 0.05% for MSMD (119). This same allele was also shown to protect against autoimmune diseases (120, 121). Homozygous carriers of this allele are not yet considered to have an IEI, but these studies suggest that susceptibility to common infections can be caused by relatively frequent AR disorders in a proportion of patients and that this outcome comes with the upside of protection from autoimmunity. Increasing numbers of disorders on the borderline between rare and common or that between monogenic and polygenic are likely to be identified as sequencing databases expand.

Most of the genetic lesions discovered in IEIs were made by whole-exome sequencing (WES). This technique is limited to assessment of the coding part of the genome. As whole-genome sequencing (WGS) gradually becomes cheaper, we are poised to discover noncoding variants with strong effects on characterized disease-causing genes. To date, fewer than a few tens of IEIs have been shown to be associated with pathogenic variants in the noncoding genome—and most of these variants are located proximal to exons (122, 123, 124). Compound heterozygosity in which a coding sequence variant interacts with a noncoding cis regulatory variant to cause an IEI have been documented, and this remains an underexplored concept (123). Further analyses of whole-genome sequence databases should not only identify increasing numbers of causal variants in regulatory regions but should also reveal noncoding modifier alleles in cis and trans that modify the expression of just the WT or other known pathological variants, thereby modifying the transcriptotype and regulating penetrance. Finally, we hypothesize that there are also protective noncoding variants that can rescue aberrant biological features when present in cis or in trans. Given the complexity of these interactions, their discovery is likely to prove difficult, but not impossible.

Studies of copy number variants (CNVs) have fallen out of fashion with the replacement of single nucleotide polymophism (SNP) arrays by WES. Only with WGS advances allowing better CNV calls will this line of research return to the fore. A few global and site-specific CNVs have already been linked to IEIs (125, 126, 127, 128, 129). However, the impact of CNVs on penetrance remains unexplored and will require cutting-edge studies technically equipped to capture large structural variations.

We think that these mechanisms are probably only the tip of the iceberg, but they will nevertheless help us to unravel the truth about incomplete penetrance.

## Principle III: Environmental exposures

### What is known

Differences in environmental exposure are frequently highlighted as putative explanations for incomplete penetrance, albeit with limited evidence. The combined effects of the environment—often referred to as the “exposome”—constitute an area of active research extending well beyond the issue of penetrance. The exposome encompasses many factors relevant to the immune system, including infections, resident microbes, diet/metabolism, irradiation, air quality, injury, and sun exposure. Many of these environmental factors are sufficient to trigger a secondary immunodeficiency in previously healthy individuals (130). However, our knowledge of the effects of environmental modifiers on penetrance in IEIs remains very limited.

Environmental factors are most easily understood in the case of susceptibility to infection. Put simply, individuals harboring variants that confer susceptibility to specific pathogens do not present disease if they are never exposed to the pathogen concerned. This is most readily appreciated in individuals with variants linked to BCG disease who do not develop disease if they are not vaccinated with BCG (131). By definition, if a susceptible individual does not encounter the infectious agent to which they are susceptible, they cannot become sick.

One very nice example in which the environment (although it is difficult to prove causality) may have contributed to incomplete penetrance is deficiencies of TIRAP, a critical adapter in TLR-based sensing. Despite having a complete innate immune defect, only one in eight TIRAP-deficient homozygotes studied presented staphylococcal disease. In the other seven, acquired anti-lipoteichoic acid antibodies (LTA Abs) (staphylococcal LTA Abs) rescued TLR-dependent susceptibility to *Staphylococcus* (132). The idea that adaptive immune responses can compensate each other is well documented for invasive pneumococcal disease due to deficiencies of IRAK4 and MyD88. Immunity changes with age, and age is known to be a major determinant of disease. Penetrance is highest at the age of 10 years, but rates of invasive pneumococcal disease recurrence and mortality fall with age, presumably due to acquired antipneumococcal immunity (133). Paradoxically, this example suggests that the very environmental exposures thought to trigger clinical presentations can also be protective.

Infection may also worsen immune dysregulation after the acute infection phase. It is now generally accepted that pathogen infections are often the event triggering autoimmune and autoinflammatory disorders. This notion is illustrated well by familial hemophagocytic lymphohistiocytosis (HLH), a disease characterized by excessive macrophage and lymphocyte activity that used to be fatal. Individuals with disease-causing variants display this hallmark cellular dysfunction early in life, before the development of clinical disease (56). Furthermore, upper respiratory or gastrointestinal tract infections tend to occur at about the onset of HLH (134). This suggests that an infectious trigger may be required for the disease to occur. Variable disease presentations are, thus, a function of exposure to an infectious agent. Further detailed documentation of the type of infection and the exact time between infection and disease onset will probably unravel certain aspects of incomplete penetrance.

Other environmental factors, such as irradiation and chemotherapy, can also modulate penetrance in IEIs. Individuals with *LIG4* variants, who have DNA repair defects leading to lymphocyte deficiencies and nonimmune features, are often healthy until treated with chemotherapy and radiotherapy. Asymptomatic carriers of *LIG4* variants have therefore probably accumulated two few double-strand breaks to cross the threshold for the development of disease (135). Studies in animals are beginning to provide experimental documentation of such effects, as shown in *Neil3*-deficient mice (97). Another example is provided by Schimke immune-osseous dysplasia, in which reduced penetrance occurs and is not sufficiently accounted for by biallelic variants of *SMARCAL1*, a conserved chromatin regulator (136, 137, 138). Studies of *Drosophila* and murine models of SMARCAL1 deficiency have suggested that an additional environmental or genetic trigger is required for full disease development (139).

### What remains unknown and future avenues for research

Immunization with live vaccines both provides answers and raises questions about the role of environmental exposures in variable penetrance. BCG vaccination is a particularly good example, as all individuals are inoculated with an identical pathogen at a very similar age, but we still observe incomplete penetrance, which is estimated at about 70% in individuals with IL12RB1 deficiency, suggesting that simple environmental differences alone cannot entirely account for this incomplete penetrance (32). Likewise, some IEI-specific pathogens are almost ubiquitous. This is the case for herpes simplex encephalitis (HSE), a sporadic disease with known monogenic etiologies (140, 141). Despite having almost complete cellular defects of TLR3-dependent IFN immunity, 4/6 TRIF-deficient, 2/3 UNC-93B-deficient, 3/8 TLR3-deficient, 3/4 IRF3-mutant, and 2/3 TBK1-hypomorphic individuals have been reported to have developed HSE (142, 143, 144, 145, 146, 147, 148, 149).

In these cases, incomplete penetrance may instead be a function of other factors, including age at exposure. In support of a major role for age in determining penetrance, HSE patients are mostly young and recurrence is rare (140). In this context, age is essentially simply a reflection of the time to first infection. What if the tonic type I IFN self-maintenance of neurons shown to be a hallmark of TLR3 deficiency was not constant but increases while oscillating in a sinusoid manner with development? If this were proven to be the case, it would provide an explanation for differences in penetrance at an early age, when HSE manifests if HSV-1 infection occurs at a upward or downward point in sinusoid type I IFN production, but not at the peak. This paradigm of increasing sinusoid type I IFN production would also help explain waning penetrance with age (143, 147, 150). This mechanism would be independent of the adaptive immune system, as HSE is not a phenotype of individuals born without adaptive immunity. In other instances of susceptibility to viruses and bacteria that are not neurotropic, prior exposure may, to a greater extent, effectively immunize the individual and regulate disease penetrance. We suggest that asymptomatic carriers of variants may have previously been exposed to noninfectious or exceedingly low doses of a pathogen, insufficient for productive infection but sufficient to induce adaptive immune responses capable of neutralizing future challenges that are truly infectious. A similar effect may occur in IL12RB1 deficiency, as the patients that develop BCG disease and those with environmental mycobacteriosis tend to form two mutually exclusive groups, suggesting that exposure to one pathogen may immunize against the other (79, 80). Similar mechanisms may operate in other susceptibilities to infection, but additional experimental evidence is required to demonstrate this.

Commensal organisms, such as the bacteria, fungi, and viruses, that naturally colonize our tissues, may be of the utmost importance. The microbiome is our most abundant source of exposure to microbes, and our symbiotic relationship to the microbiome is therefore of considerable importance. Early experiments in which the gut bacterial microbiome was eliminated with a cocktail of antibiotics ultimately resulted in higher levels of inflammation than in untreated animals, suggesting a true homeostatic function. In the last 2 decades, the microbiome has proved a major determinant of immune function and disease (151, 152). Despite these strong associations, the relevance of the microbiome in IEIs—the most extreme immune system diseases—remains unknown. Recent studies demonstrating changes to the bacterial microbiota in CVID and their correlation with immune activation and certain symptoms have begun to scratch the surface, but the direction of causality remains unclear (153, 154, 155). We suggest that the bacterial, viral, and fungal biota regulates the penetrance of IEIs by shaping relative innate and/or adaptive tolerance and reactivity. The divergence of the microbiome with geography and diet may underlie the variability of IEI phenotypes across populations with similar monogenic lesions. Detailed future studies are required to document the types and quantities of microbiota in IEI cohorts.

Our environment, which is continuing to change, is very different from that in which our ancestral immune system evolved. Six years ago, SARS-CoV2 was not present, and just a century ago, 30–50% of us would not have lived beyond early childhood, as death from infection was 200 times more frequent (156). It seems likely that a study on the genetics of infectious disease a century ago, with the tools of today, would have identified far more common alleles as casual. However, today, these genetic susceptibilities, which we refer to as common variants, are probably masked by the protective effects of good sanitation, vaccination, and antibiotic use. Perhaps we should consider all these potential susceptibility alleles in isolated systems (as we have mapped the key genes), as this would undoubtedly be informative and improve our understanding of incomplete penetrance. Conversely, the recent development of immunosuppressant use in transplantation, immunology, rheumatology, dermatology, and neurology may reveal new and old genetic susceptibilities with surprising frequencies and pathogen specificities.

## Principle IV: The mosaicism of disease-causing alleles reduces clinical penetrance

### What is known

But it is even more complicated than that. Up to this point in the discussion, we have assumed that all the cells in an affected individual carry the same variant. However, genetic differences between cells occur at a surprisingly high frequency within individuals. Genetic mosaicism originates from post-zygotic (de novo) variants that arise during the embryonic or postnatal period. The occurrence of such mosaicism in IEIs was initially thought to be rare, but it has since been found to be rather common. A recent systematic analysis across IEIs by targeted deep sequencing in 128 families estimated the rate of mosaicism at 23.4% (157). In the last 5 years alone, the number of mutated genes with mosaicism shown to cause IEIs has almost doubled, bringing the count to over 20 (158, 159). Interestingly, some of these variants cause disease in the mosaic state, as opposed to the mosaic and germline states, presumably due to a strong germline impact.

Disease onset and/or severity are variable in cases of mosaicism, as a direct consequence of gene dosage, the tissue affected, and time since somatic variant generation. Incomplete penetrance in mosaic IEIs was first documented in an extraordinary case of delayed-onset ADA deficiency, which is typically a severe form of SCID, during the 1980s and 1990s. *ADA* mosaicism was observed directly in peripheral blood cells and ADA-normal populations gradually came to predominate over time, with the resolution of clinical disease (160, 161, 162). After this discovery, several other documented cases of disease-associated mosaicism were reported (163, 164), some presenting as mild or atypical disease phenotypes, including variants of *NLRP3* (165, 166), *STAT3* (167,168), *FAS* (169, 170), *CYBB* (171), *TNFAIP3* (172), TRAPS (173), IL6ST (174), TLR8 (175), and RAP1B (176). It should be noted that all of these cases predominantly concern disorders of immune hyperactivation rather than deficiency.

As the number of such cases has grown, the evidence for mosaicism and pseudogene dosage as a mechanism underlying reduced penetrance has also increased, with an apparently good correlation. In an analysis of 10 families in which one member carried a postzygotic IEI gene variant, 80% of the mosaic individuals were asymptomatic. The remaining mosaic individuals presented with only partial clinical disease, whereas their progeny with an inherited germline variant displayed full disease development (157). An evaluation of variant read frequencies in a family with *PIK3CD* variants revealed that affected siblings harbored more mutant cells than their mildly affected father, with allele fractions of 37–54% and 15%, respectively (16). Conversely, if mutated cells predominate in the relevant cell compartment, the clinical features of germline and somatic variant are more similar. ALPS patients harboring FAS variants in ∼100% of their DN T cells display complete disease development despite having undetectable levels of the variant in whole blood (169, 170).

In the realm of new somatic variants of genes causing IEIs that have never been described in the germline, the best example is probably that of *UBA1* variants causing vacuoles, E1-ubiquitin-activating enzyme, X-linked, autoinflammatory, somatic (VEXAS) syndrome (177). The clinical signs of VEXAS syndrome overlap strongly with those of giant cell arteritis, relapsing polychondritis, systemic lupus erythematosus (SLE), and rheumatoid arthritis (RA). Since its discovery only 5 years ago, hundreds of patients have been identified, with extremely diverse clinical disease penetrance, despite the presence of exactly the same variants in most of these patients (178).

Somatic variants may act as modifiers and, thus, as “second hits” leading to the manifestation of clinical disease. ALPS patients have been documented to carry both an inherited heterozygous *FAS* variant and a somatic event in the second *FAS* allele, such as a missense variant, nonsense variant, or loss of heterozygosity (179, 180, 181). Alternatively, the second hit may occur at a different locus, as in a recent report of a somatic *FAS* variant occurring together with an existing *CASP10* variant (182). Relatives who did not acquire a second variant post-zygotically remained asymptomatic or were only partially affected, suggesting an effect of second hit mosaicism on incomplete penetrance.

Conversely, the acquisition of a somatic variant can also rescue disease. Such events, often referred to as somatic reversions, underlie milder clinical disease or the absence of clinical disease. A good example is provided by the reversion of DOCK-8 deficiency, which is commonly, occurring in about half of all affected patients; this reversion is associated with longer survival and less severe allergic disease, although preliminary reports have suggested that infectious disease susceptibility remains the same (183). Full recovery from disease, including infectious phenotypes was reported in a more recent study (184). There are several other examples of reversions underlying incompletely penetrant clinical disease, for ADA (162, 185), XLA (186, 187), WASP (188, 189), leukocyte adhesion deficiency (190), X-linked immunodeficiency with ectodermal dysplasia due to variants in *NEMO* (191), Omenn syndrome with *CARD11* deficiency (192), IKBKG-associated immunodeficiency (16), and GATA2 deficiency (193). Interestingly, these reversions may even involve second-site variants in the initially mutated gene creating altered non-WT, but still functional, gene products (194). Reversion may also occur via chromosomal and segmental chromosomal deletions, as shown for the *SAMD9* and *SAMD9L* alleles, for which this reversion occurs via monosomy 7 (195). Reversions, thus, represent a common and complex component of incomplete penetrance.

Most mosaic IEIs appear to remain stable over time (157, 166). However, somatic reversions conferring a fitness advantage may enable the selective expansion of the reverted cell population to reestablish healthy immune cell populations. Documented reversions of variants of *JAK3*, an essential mediator of lymphocyte development, can repair immune cell proliferation and differentiation. In one family with JAK3 hypomorphic variants, the asymptomatic sibling displayed CD4^+^ T cell reversion, whereas a brother without this reversion suffered recurrent respiratory tract infections (196). One fascinating case was reported in a warts, hypogammaglobulinemia, infections, and myelokathexis (WHIM) syndrome patient cured by a process known as chromothripsis, or “chromosome shattering,” in which the chromosomes undergo massive deletion and rearrangement. Fortuitously, this event deleted the mutated *CXCR4* allele in a single hematopoietic stem cell, which then took over the bone marrow and reconstituted immune function (197). By contrast, if there is no selective pressure due to treatment, as in enzyme replacement therapy in ADA deficiency with reversions or allogeneic stem cell therapy, the WT cells appear to lose their selective advantage and their proportions decline (188). This example raises the question as to how best to help patients to help themselves.

### What remains unknown and future avenues for research

Mosaicism usually tempers the penetrance of its germline counterpart, but there are cases in which mosaic variants lead to an equally or even more severe disease (8, 157, 195, 198, 199). Of course, the same could be said of the first and only reports of patients with mosaic variants for which there is no germline counterpart, suggesting that germline defects may be lethal at the embryonic or perinatal stages (158, 168, 200, 201, 202, 203). The application of more recent technologies to larger cohorts and improvements in tissue sampling will be essential to address the remaining questions. Low-frequency somatic variants probably still escape most detection approaches. It is important to solve this problem as mosaic variants present even at a frequency of 0.5% of tissue can cause disease. This frequency is not simply a function of total mosaic fractions but also depends on the tissue analyzed. Many somatic variants are only detectable in specific immune cell types (158, 165, 168, 170, 178), and many more such cell type–specific variants than are currently known are likely to exist. Perhaps the most underexplored variants are extrahematopoietic variants, probably due to difficulties with tissue sampling.

As discussed above, going beyond the genotype, mosaicism can also exist at the transcript level across genetically identical cells in which one autosome is more transcriptionally active than the other due to RMAE (204, 205, 206, 207, 208, 209). This de facto transcriptional mosaicism can occur on top of genetic mosaicism, which we initially demonstrated in 2020 (210). Remarkably, up to 10% of the autosomal genome displays this phenomenon (204).

For these genes, allelic bias (whether a germline or somatic variant) is established in lineage differentiation via a unique chromatin signature, DNA methylation, and persists during subsequent cell divisions (85, 211, 212). Contrasting with the situation only 5 years ago, we are now increasingly able to understand the nature of this epigenetic phenomenon, which can occur on mosaic background as well. We are beginning to grasp the functional consequences, especially in light of genetic disease and penetrance. Computational predictions have suggested that there is an enrichment in monoallelic expression (MAE) among genes for which gain-of-function variants with AD inheritance have been linked to neuropsychiatric disease (213) Disease-related genes have been shown, experimentally, to undergo MAE (214) and, in 2020, the first gene variant with an allelic bias was documented in a mosaic patient with *JAK1* variant (8). Earlier this year, we showed that MAE can account for disease penetrance in the members of families with *JAK1*, *STAT1*, *CARD11*, or *PLCG2* variants (85). Beyond JAK1, it remains to be seen if MAE occurs in other mosaic patients.

Heterozygosity for variants of genes displaying MAE genes, thus, create a mixture of WT- and mutant-expressing cells with divergent phenotypes in affected individuals. We have now shown that, by creating this mosaic transcriptotype, MAE can modulate the functional impact of disease-causing variants in various ways and proportions (8, 85). MAE no longer a hypothesis can actually help explain phenotypic variation in genetic disease. In AD disease, mosaicism reduces the penetrance of disease phenotypes in patients. In AR disease, this phenomenon is predicted to occur in affected carriers but has not yet been experimentally demonstrated. We have shown that up to 4% of IEI genes can undergo MAE in healthy individuals. It remains unknown whether variant can itself drive MAE, but it may increase the proportion of genes capable of displaying MAE, perhaps to 30–50% of all IEI genes. It remains unclear whether MAE accounts for only a minority of cases with incomplete penetrance or whether the documented cases are just the tip of the iceberg, which on mosaic background will be very exciting to further document.

## Conclusions

**Figure 1.**
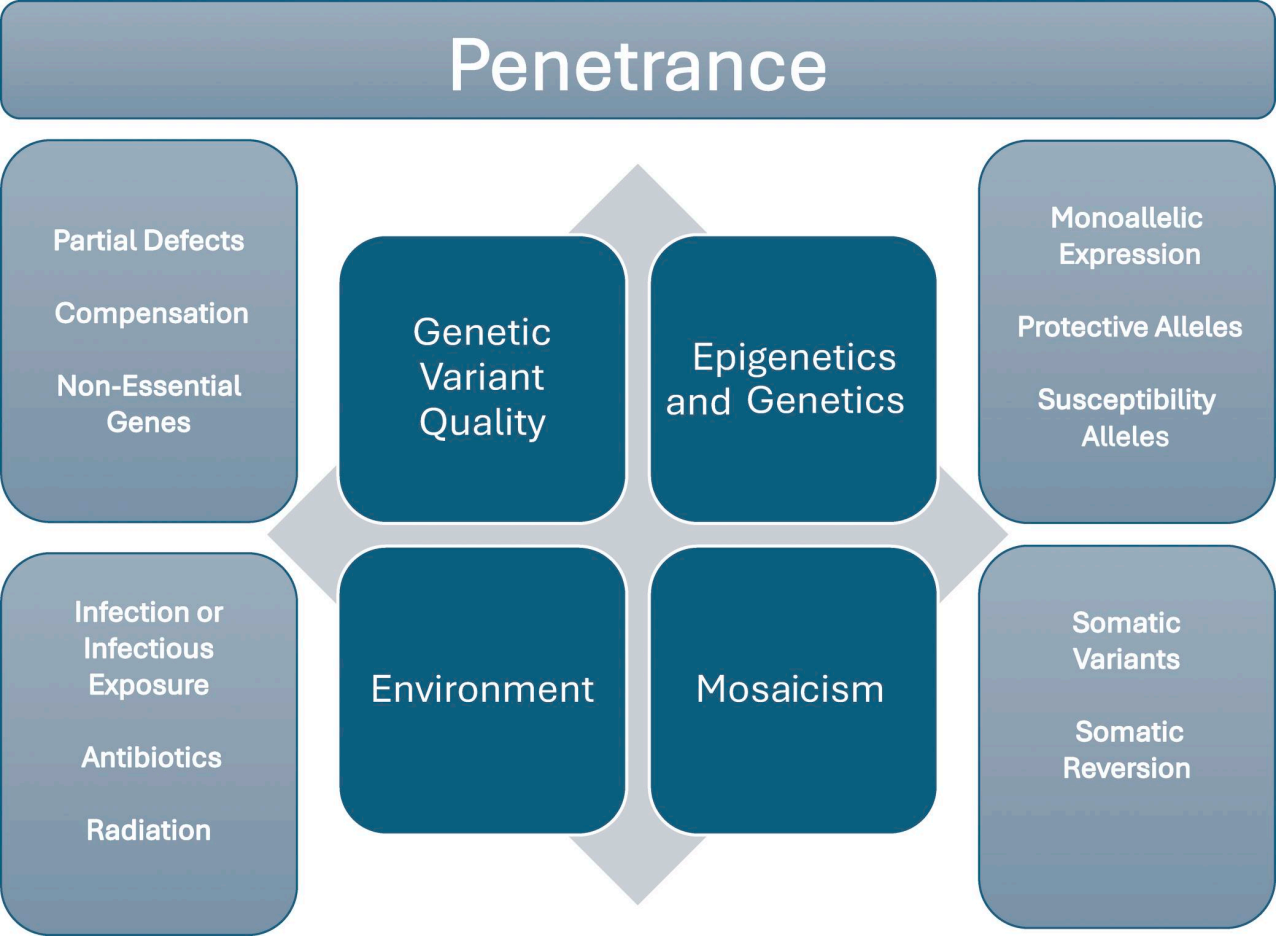
Graphical representation of four incomplete penetrance principles.

Not understanding penetrance in IEIs, and indeed in all genetic diseases, has hindered advances in human genetics. By documenting and classifying the cases of variable penetrance in IEIs, this review, like its predecessor (25), aims to shed light on the existing connections and the persistent gaps in our knowledge. It is clear that four major influences continually reduce penetrance— genetic variant quality, epigenetic and genetic modifiers, environmental influences, and mosaicism—whereas many aspects of these four principles, such as genomic compensation, protective variants, subinfectious inoculations, monoallelic expression, and peripheral tissue mosaics, remain unexplored (Fig. 1). We mostly discuss these principles separately here, but they do work in tandem and interact, as biology and medicine do not self-classify. We impose classifications to ensure clarity. The key breakthroughs in these domains do not come from single sources, but from the combined efforts of large cohorts, intense studies of single patients, model organisms, and even cell lines. It is important to keep an open mind, as many more natural laws remain to be discovered, and there are undoubtedly surprises hiding in plain sight. Furthering our understanding of penetrance will therefore continue to require both an open mind and rigorous studies.

## References

[1] Wiskott, A. 1937. Familiärer, angeborener Morbus Werlhofii? Monatsschr Kinderheilkd. 68.

[2] Lutz, W. 1946. A propos de l’Epidermodysplasie verruciforme. Dermatology. 92:30–43. 20982046

[3] Kostmann, R. 1950. Hereditär reticulos-en ny systemsjukdom. Svenska Läkartideningen. 47:2861–8-2861.

[4] Bruton, O. 1952. Agammaglobulinemia. Pediatrics. 9:722–728. 14929630

[5] Aldrich, R.A., A.G. Steinberg, and D.C. Campbell. 1954. Pedigree demonstrating a sex-linked recessive condition characterized by draining ears, eczematoid dermatitis and bloody diarrhea. Pediatrics. 13:133–139. 13133561

[6] Poli, M.C., I. Aksentijevich, A.A. Bousfiha, C. Cunningham-Rundles, S. Hambleton, C. Klein, T. Morio, C. Picard, A. Puel, N. Rezaei, . 2025. Human inborn errors of immunity: 2024 update on the classification from the international union of immunological societies expert committee. J. Hum. Immun. 1:e20250003. 10.70962/jhi.20250003 41608114 PMC12829761

[7] Taft, J., M. Markson, D. Legarda, R. Patel, M. Chan, L. Malle, A. Richardson, C. Gruber, M. Martín-Fernández, G.M.S. Mancini, . 2021. Human TBK1 deficiency leads to autoinflammation driven by TNF-induced cell death. Cell. 184:4447–4463.e20. 10.1016/j.cell.2021.07.026 34363755 PMC8380741

[8] Gruber, C.N., J.J.A. Calis, S. Buta, G. Evrony, J.C. Martin, S.A. Uhl, R. Caron, L. Jarchin, D. Dunkin, R. Phelps, . 2020. Complex autoinflammatory syndrome unveils fundamental principles of JAK1 kinase transcriptional and biochemical function. Immunity. 53:672–684 e11. 10.1016/j.immuni.2020.07.006 32750333 PMC7398039

[9] Alsohime, F., M. Martin-Fernandez, M.-H. Temsah, M. Alabdulhafid, T. Le Voyer, M. Alghamdi, X. Qiu, N. Alotaibi, A. Alkahtani, S. Buta, . 2020. JAK inhibitor therapy in a child with inherited USP18 deficiency. N. Engl. J. Med. 382:256–265. 10.1056/NEJMoa1905633 31940699 PMC7155173

[10] Delavari, S., S.E. Rasouli, S. Fekrvand, Z. Chavoshzade, S.A. Mahdaviani, P. Shirmast, S. Sharafian, R. Sherkat, T. Momen, S. Aleyasin, . 2024. Clinical heterogeneity in families with multiple cases of inborn errors of immunity. Clin. Immunol. 259:109896. 10.1016/j.clim.2024.109896 38184287

[11] Yel, L. 2010. Selective IgA deficiency. J. Clin. Immunol. 30:10–16. 10.1007/s10875-009-9357-x 20101521 PMC2821513

[12] Kong, X.-F., G. Vogt, Y. Itan, A. Macura-Biegun, A. Szaflarska, D. Kowalczyk, A. Chapgier, A. Abhyankar, D. Furthner, C. Djambas Khayat, . 2013. Haploinsufficiency at the human IFNGR2 locus contributes to mycobacterial disease. Hum. Mol. Genet. 22:769–781. 10.1093/hmg/dds484 23161749 PMC3554203

[13] Lorenzini, T., M. Fliegauf, N. Klammer, N. Frede, M. Proietti, A. Bulashevska, N. Camacho-Ordonez, M. Varjosalo, M. Kinnunen, E. de Vries, . 2020. Characterization of the clinical and immunologic phenotype and management of 157 individuals with 56 distinct heterozygous NFKB1 mutations. J. Allergy Clin. Immunol. 146:901–911. 10.1016/j.jaci.2019.11.051 32278790 PMC8246418

[14] Vihinen, M., B.H. Belohradsky, R.N. Haire, E. Holinski-Feder, S.P. Kwan, I. Lappalainen, H. Lehväslaiho, T. Lester, A. Meindl, H.D. Ochs, . 1997. BTKbase, mutation database for X-linked agammaglobulinemia (XLA). Nucleic Acids Res. 25:166–171. 10.1093/nar/25.1.166 9016530 PMC146405

[15] Candotti, F. 2018. Clinical manifestations and pathophysiological mechanisms of the Wiskott-Aldrich syndrome. J. Clin. Immunol. 38:13–27. 10.1007/s10875-017-0453-z 29086100

[16] Stray-Pedersen, A., H.S. Sorte, P. Samarakoon, T. Gambin, I.K. Chinn, Z.H. Coban Akdemir, H.C. Erichsen, L.R. Forbes, S. Gu, B. Yuan, . 2017. Primary immunodeficiency diseases: Genomic approaches delineate heterogeneous Mendelian disorders. J. Allergy Clin. Immunol. 139:232–245. 10.1016/j.jaci.2016.05.042 27577878 PMC5222743

[17] Gudmundsson, S., M. Singer-Berk, N.A. Watts, W. Phu, J.K. Goodrich, M. Solomonson, Genome Aggregation Database Consortium, H.L. Rehm, D.G. MacArthur, and A. O’Donnell-Luria. 2022. Variant interpretation using population databases: Lessons from gnomAD. Hum. Mutat. 43:1012–1030. 10.1002/humu.24309 34859531 PMC9160216

[18] Walsh, R., K.L. Thomson, J.S. Ware, B.H. Funke, J. Woodley, K.J. McGuire, F. Mazzarotto, E. Blair, A. Seller, J.C. Taylor, . 2017. Reassessment of Mendelian gene pathogenicity using 7,855 cardiomyopathy cases and 60,706 reference samples. Genet. Med. 19:192–203. 10.1038/gim.2016.90 27532257 PMC5116235

[19] Chen, R., L. Shi, J. Hakenberg, B. Naughton, P. Sklar, J. Zhang, H. Zhou, L. Tian, O. Prakash, M. Lemire, . 2016. Analysis of 589,306 genomes identifies individuals resilient to severe Mendelian childhood diseases. Nat. Biotechnol. 34:531–538. 10.1038/nbt.3514 27065010

[20] Cooper, D.N., M. Krawczak, C. Polychronakos, C. Tyler-Smith, and H. Kehrer-Sawatzki. 2013. Where genotype is not predictive of phenotype: Towards an understanding of the molecular basis of reduced penetrance in human inherited disease. Hum. Genet. 132:1077–1130. 10.1007/s00439-013-1331-2 23820649 PMC3778950

[21] Walsh, R., R. Tadros, and C.R. Bezzina. 2020. When genetic burden reaches threshold. Eur. Heart J. 41:3849–3855. 10.1093/eurheartj/ehaa269 32350504 PMC7599032

[22] Kingdom, R. and C.F. Wright. 2022. Incomplete penetrance and variable expressivity: From clinical studies to population cohorts*.* Front. Genet. 13:920390. 10.3389/fgene.2022.920390 35983412 PMC9380816

[23] Ciesielski, T.H., G. Sirugo, S.K. Iyengar, and S.M. Williams. 2024. Characterizing the pathogenicity of genetic variants: The consequences of context. NPJ Genom. Med. 9:3. 10.1038/s41525-023-00386-5 38195641 PMC10776585

[24] Wright, C.F., L.N. Sharp, L. Jackson, A. Murray, J.S. Ware, D.G. MacArthur, H.L. Rehm, K.A. Patel, and M.N. Weedon. 2024. Guidance for estimating penetrance of monogenic disease-causing variants in population cohorts. Nat. Genet. 56:1772–1779. 10.1038/s41588-024-01842-3 39075210

[25] Gruber, C., and D. Bogunovic. 2020. Incomplete penetrance in primary immunodeficiency: A skeleton in the closet. Hum. Genet. 139:745–757. 10.1007/s00439-020-02131-9 32067110 PMC7275875

[26] Jouanguy, E., F. Altare, S. Lamhamedi, P. Revy, J.F. Emile, M. Newport, M. Levin, S. Blanche, E. Seboun, A. Fischer, and J.L. Casanova. 1996. Interferon-γ –receptor deficiency in an infant with fatal bacille Calmette–Guérin infection. New Engl. J. Med. 335:1956–1962. 10.1056/NEJM199612263352604 8960475

[27] Newport, M.J., C.M. Huxley, S. Huston, C.M. Hawrylowicz, B.A. Oostra, R. Williamson, and M. Levin. 1996. A mutation in the interferon-gamma-receptor gene and susceptibility to mycobacterial infection. N. Engl. J. Med. 335:1941–1949. 10.1056/NEJM199612263352602 8960473

[28] Bustamante, J., S. Boisson-Dupuis, L. Abel, and J.-L. Casanova. 2014. Mendelian susceptibility to mycobacterial disease: Genetic, immunological, and clinical features of inborn errors of IFN-γ immunity. Semin. Immunol. 26:454–470. 10.1016/j.smim.2014.09.008 25453225 PMC4357480

[29] Rosain, J., X.-F. Kong, R. Martinez-Barricarte, C. Oleaga-Quintas, N. Ramirez-Alejo, J. Markle, S. Okada, S. Boisson-Dupuis, J.L. Casanova, and J. Bustamante. 2019. Mendelian susceptibility to mycobacterial disease: 2014–2018 update. Immunol. Cell Biol. 97:360–367. 10.1111/imcb.12210 30264912 PMC6438774

[30] Jouanguy, E., S. Lamhamedi-Cherradi, D. Lammas, S.E. Dorman, M.C. Fondanèche, S. Dupuis, R. Döffinger, F. Altare, J. Girdlestone, J.F. Emile, . 1999. A human IFNGR1 small deletion hotspot associated with dominant susceptibility to mycobacterial infection. Nat. Genet. 21:370–378. 10.1038/7701 10192386

[31] Dorman, S.E., C. Picard, D. Lammas, K. Heyne, J.T. van Dissel, R. Baretto, S.D. Rosenzweig, M. Newport, M. Levin, J. Roesler, . 2004. Clinical features of dominant and recessive interferon γ receptor 1 deficiencies. Lancet. 364:2113–2121. 10.1016/S0140-6736(04)17552-1 15589309

[32] Bustamante, J., S. Boisson-Dupuis, L. Abel, and J.-L. Casanova. 2014. Mendelian susceptibility to mycobacterial disease: Genetic, immunological, and clinical features of inborn errors of IFN-γ immunity. Semin. Immunol. 26:454–470. 10.1016/j.smim.2014.09.008 25453225 PMC4357480

[33] Dupuis, S., E. Jouanguy, S. Al-Hajjar, C. Fieschi, I.Z. Al-Mohsen, S. Al-Jumaah, K. Yang, A. Chapgier, C. Eidenschenk, P. Eid, . 2003. Impaired response to interferon-alpha/beta and lethal viral disease in human STAT1 deficiency. Nat. Genet. 33:388–391. 10.1038/ng1097 12590259

[34] Chapgier, A., S. Boisson-Dupuis, E. Jouanguy, G. Vogt, J. Feinberg, A. Prochnicka-Chalufour, A. Casrouge, K. Yang, C. Soudais, C. Fieschi, . 2006. Novel STAT1 alleles in otherwise healthy patients with mycobacterial disease. PLoS Genet. 2:e131. 10.1371/journal.pgen.0020131 16934001 PMC1550284

[35] Vairo, D., L. Tassone, G. Tabellini, N. Tamassia, S. Gasperini, F. Bazzoni, A. Plebani, F. Porta, L.D. Notarangelo, S. Parolini, . 2011. Severe impairment of IFN-γ and IFN-α responses in cells of a patient with a novel STAT1 splicing mutation. Blood. 118:1806–1817. 10.1182/blood-2011-01-330571 21772053

[36] Chapgier, A., X.-F. Kong, S. Boisson-Dupuis, E. Jouanguy, D. Averbuch, J. Feinberg, S.Y. Zhang, J. Bustamante, G. Vogt, J. Lejeune, . 2009. A partial form of recessive STAT1 deficiency in humans. J. Clin. Invest. 119:1502–1514. 10.1172/JCI37083 19436109 PMC2689115

[37] Kong, X.-F., M. Ciancanelli, S. Al-Hajjar, L. Alsina, T. Zumwalt, J. Bustamante, J. Feinberg, M. Audry, C. Prando, V. Bryant, . 2010. A novel form of human STAT1 deficiency impairing early but not late responses to interferons. Blood. 116:5896–5906. 10.1182/blood-2010-04-280586 PMC3031383 20841510

[38] Kristensen, I.A., J.E. Veirum, B.K. Møller, and M. Christiansen. 2011. Novel STAT1 alleles in a patient with impaired resistance to mycobacteria. J. Clin. Immunol. 31:265–271. 10.1007/s10875-010-9480-8 21057861

[39] Chapgier, A., R.F. Wynn, E. Jouanguy, O. Filipe-Santos, S. Zhang, J. Feinberg, K. Hawkins, J.-L. Casanova, and P.D. Arkwright. 2006. Human complete Stat-1 deficiency is associated with defective type I and II IFN responses in vitro but immunity to some low virulence viruses in vivo. J. Immunol. 176:5078–5083. 10.4049/jimmunol.176.8.5078 16585605

[40] Sampaio, E.P., H.I. Bax, A.P. Hsu, E. Kristosturyan, J. Pechacek, P. Chandrasekaran, M.L. Paulson, D.L. Dias, C. Spalding, G. Uzel, . 2012. A novel STAT1 mutation associated with disseminated mycobacterial disease. J. Clin. Immunol. 32:681–689. 10.1007/s10875-012-9659-2 22437822 PMC4112946

[41] Tsumura, M., S. Okada, H. Sakai, S. Yasunaga, M. Ohtsubo, T. Murata, H. Obata, T. Yasumi, X.-F. Kong, A. Abhyankar, . 2012. Dominant-negative STAT1 SH2 domain mutations in unrelated patients with mendelian susceptibility to mycobacterial disease. Hum. Mutat. 33:1377–1387. 10.1002/humu.22113 22573496 PMC3668973

[42] Minegishi, Y., M. Saito, S. Tsuchiya, I. Tsuge, H. Takada, T. Hara, N. Kawamura, T. Ariga, S. Pasic, O. Stojkovic, . 2007. Dominant-negative mutations in the DNA-binding domain of STAT3 cause hyper-IgE syndrome. Nature. 448:1058–1062. 10.1038/nature06096 17676033

[43] Hambleton, S., S. Goodbourn, D.F. Young, P. Dickinson, S.M.B. Mohamad, M. Valappil, N. McGovern, A.J. Cant, S.J. Hackett, P. Ghazal, . 2013. STAT2 deficiency and susceptibility to viral illness in humans. Proc. Natl. Acad. Sci. USA. 110:3053–3058. 10.1073/pnas.1220098110 23391734 PMC3581986

[44] Duncan, C.J.A., S.M.B. Mohamad, D.F. Young, A.J. Skelton, T.R. Leahy, D.C. Munday, K.M. Butler, S. Morfopoulou, J.R. Brown, M. Hubank, . 2015. Human IFNAR2 deficiency: Lessons for antiviral immunity. Sci. Transl. Med. 7:307ra154. 10.1126/scitranslmed.aac4227 PMC4926955 26424569

[45] Kreins, A.Y., M.J. Ciancanelli, S. Okada, X.-F. Kong, N. Ramírez-Alejo, S.S. Kilic, J. El Baghdadi, S. Nonoyama, S.A. Mahdaviani, F. Ailal, . 2015. Human TYK2 deficiency: Mycobacterial and viral infections without hyper-IgE syndrome. J. Exp. Med. 212:1641–1662. 10.1084/jem.20140280 26304966 PMC4577846

[46] Moens, L., L. Van Eyck, D. Jochmans, T. Mitera, G. Frans, X. Bossuyt, P. Matthys, J. Neyts, M. Ciancanelli, S.Y. Zhang, . 2017. A novel kindred with inherited STAT2 deficiency and severe viral illness. J. Allergy Clin. Immunol. 139:1995–1997.e9. 10.1016/j.jaci.2016.10.033 28087227

[47] Sarrafzadeh, S.A., M. Mahloojirad, J.-L. Casanova, M. Badalzadeh, J. Bustamante, S. Boisson-Dupuis, Z. Pourpak, M. Nourizadeh, and M. Moin. 2020. A new patient with inherited TYK2 deficiency. J. Clin. Immunol. 40:232–235. 10.1007/s10875-019-00713-5 31713088 PMC7218688

[48] Bastard, P., J. Manry, J. Chen, J. Rosain, Y. Seeleuthner, O. AbuZaitun, L. Lorenzo, T. Khan, M. Hasek, N. Hernandez, . 2021. Herpes simplex encephalitis in a patient with a distinctive form of inherited IFNAR1 deficiency. J. Clin. Invest. 131:e139980. 10.1172/JCI139980 32960813 PMC7773360

[49] Meyts, I., and J.-L. Casanova. 2021. Viral infections in humans and mice with genetic deficiencies of the type I IFN response pathway. Eur. J. Immunol. 51:1039–1061. 10.1002/eji.202048793 33729549 PMC8900014

[50] Bastard, P., K.-C. Hsiao, Q. Zhang, J. Choin, E. Best, J. Chen, A. Gervais, L. Bizien, M. Materna, C. Harmant, . 2022. A loss-of-function IFNAR1 allele in Polynesia underlies severe viral diseases in homozygotes. J. Exp. Med. 219:e20220028. 10.1084/jem.20220028 35442418 PMC9026234

[51] Duncan, C.J.A., M.K. Skouboe, S. Howarth, A.K. Hollensen, R. Chen, M.L. Børresen, B.J. Thompson, J. Stremenova Spegarova, C.F. Hatton, F.F. Stæger, . 2022. Life-threatening viral disease in a novel form of autosomal recessive IFNAR2 deficiency in the Arctic. J. Exp. Med. 219:e20212427. 10.1084/jem.20212427 35442417 PMC9026249

[52] Taft, J., and D. Bogunovic. 2018. The goldilocks zone of type I IFNs: Lessons from human genetics. J. Immunol. 201:3479–3485. 10.4049/jimmunol.1800764 30530500

[53] Goel, S., H.S. Kuehn, J. Chinen, J. Niemela, J. Stoddard, D. Yamanaka, M. Garofalo, S. Samir, M. Migaud, V. Oikonomou, . 2022. CARD9 expression pattern, gene dosage, and immunodeficiency phenotype revisited. J. Clin. Immunol. 42:336–349. 10.1007/s10875-021-01173-6 34791587 PMC10108093

[54] Imanaka, Y., M. Taniguchi, T. Doi, M. Tsumura, R. Nagaoka, M. Shimomura, T. Asano, R. Kagawa, Y. Mizoguchi, S. Karakawa, . 2021. Inherited CARD9 deficiency in a child with invasive disease due to exophiala dermatitidis and two older but asymptomatic siblings. J. Clin. Immunol. 41:975–986. 10.1007/s10875-021-00988-7 33558980

[55] Jagle, S., M. Heeg, S. Grün, A. Rensing-Ehl, M.E. Maccari, C. Klemann, N. Jones, K. Lehmberg, C. Bettoni, K. Warnatz, . 2020. Distinct molecular response patterns of activating STAT3 mutations associate with penetrance of lymphoproliferation and autoimmunity*.* Clin. Immunol. 210:108316. 10.1016/j.clim.2019.108316 31770611

[56] Feldmann, J., F. Le Deist, M. Ouachée-Chardin, S. Certain, S. Alexander, P. Quartier, E. Haddad, N. Wulffraat, J.L. Casanova, S. Blanche, . 2002. Functional consequences of perforin gene mutations in 22 patients with familial haemophagocytic lymphohistiocytosis. Br. J. Haematol. 117:965–972. 10.1046/j.1365-2141.2002.03534.x 12060139

[57] Molleran Lee, S., J. Villanueva, J. Sumegi, K. Zhang, K. Kogawa, J. Davis, and A.H. Filipovich. 2004. Characterisation of diverse PRF1 mutations leading to decreased natural killer cell activity in North American families with haemophagocytic lymphohistiocytosis. J. Med. Genet. 41:137–144. 10.1136/jmg.2003.011528 14757862 PMC1735659

[58] Tanita, K., F. Sakura, R. Nambu, M. Tsumura, Y. Imanaka, H. Ohnishi, Z. Kato, J. Pan, A. Hoshino, K. Suzuki, . 2021. Clinical and immunological heterogeneity in Japanese patients with gain-of-function variants in STAT3. J. Clin. Immunol. 41:780–790. 10.1007/s10875-021-00975-y 33501615

[59] Oftedal, B.E., A. Hellesen, M.M. Erichsen, E. Bratland, A. Vardi, J. Perheentupa, E.H. Kemp, T. Fiskerstrand, M.K. Viken, A.P. Weetman, . 2015. Dominant mutations in the autoimmune regulator AIRE are associated with common organ-specific autoimmune diseases. Immunity. 42:1185–1196. 10.1016/j.immuni.2015.04.021 26084028

[60] Oftedal, B.E., K. Assing, S. Baris, S.L. Safgren, I.S. Johansen, M.A. Jakobsen, D. Babovic-Vuksanovic, K. Agre, E.W. Klee, E. Majcic, . 2023. Dominant-negative heterozygous mutations in AIRE confer diverse autoimmune phenotypes. iScience. 26:106818. 10.1016/j.isci.2023.106818 37235056 PMC10206195

[61] Bolze, A., B. Boisson, B. Bosch, A. Antipenko, M. Bouaziz, P. Sackstein, M. Chaker-Margot, V. Barlogis, T. Briggs, E. Colino, . 2018. Incomplete penetrance for isolated congenital asplenia in humans with mutations in translated and untranslated RPSA exons. Proc. Natl. Acad. Sci. USA. 115:E8007–E8016. 10.1073/pnas.1805437115 30072435 PMC6112730

[62] Rieux-Laucat, F., F. Le Deist, C. Hivroz, I.A. Roberts, K.M. Debatin, A. Fischer, and J.P. de Villartay. 1995. Mutations in Fas associated with human lymphoproliferative syndrome and autoimmunity. Science. 268:1347–1349. 10.1126/science.7539157 7539157

[63] Le Deist, F., J.F. Emile, F. Rieux-Laucat, M. Benkerrou, I. Roberts, N. Brousse, and A. Fischer. 1996. Clinical, immunological, and pathological consequences of Fas-deficient conditions. Lancet. 348:719–723. 10.1016/S0140-6736(96)02293-3 8806292

[64] Kasahara, Y., T. Wada, Y. Niida, A. Yachie, H. Seki, Y. Ishida, T. Sakai, F. Koizumi, S. Koizumi, T. Miyawaki, and N. Taniguchi. 1998. Novel Fas (CD95/APO-1) mutations in infants with a lymphoproliferative disorder. Int. Immunol. 10:195–202. 10.1093/intimm/10.2.195 9533447

[65] van der Burg, M., R. de Groot, W.M. Comans-Bitter, J.C. den Hollander, H. Hooijkaas, H.J. Neijens, R.M. Berger, A.P. Oranje, A.W. Langerak, and J.J. van Dongen. 2000. Autoimmune lymphoproliferative syndrome (ALPS) in a child from consanguineous parents: A dominant or recessive disease? Pediatr. Res. 47:336–343. 10.1203/00006450-200003000-00009 10709732

[66] Kuehn, H.S., I. Caminha, J.E. Niemela, V.K. Rao, J. Davis, T.A. Fleisher, and J.B. Oliveira. 2011. FAS haploinsufficiency is a common disease mechanism in the human autoimmune lymphoproliferative syndrome. J. Immunol. 186:6035–6043. 10.4049/jimmunol.1100021 21490157 PMC3725553

[67] Infante, A.J., H.A. Britton, T. DeNapoli, L.A. Middelton, M.J. Lenardo, C.E. Jackson, J. Wang, T. Fleisher, S.E. Straus, and J.M. Puck. 1998. The clinical spectrum in a large kindred with autoimmune lymphoproliferative syndrome caused by a Fas mutation that impairs lymphocyte apoptosis. J. Pediatr. 133:629–633. 10.1016/s0022-3476(98)70102-7 9821419

[68] Jackson, C.E., R.E. Fischer, A.P. Hsu, S.M. Anderson, Y. Choi, J. Wang, J.K. Dale, T.A. Fleisher, L.A. Middelton, M.C. Sneller, . 1999. Autoimmune lymphoproliferative syndrome with defective Fas: Genotype influences penetrance. Am. J. Hum. Genet. 64:1002–1014. 10.1086/302333 10090885 PMC1377824

[69] Bleesing, J.J., M.R. Brown, S.E. Straus, J.K. Dale, R.M. Siegel, M. Johnson, M.J. Lenardo, J.M. Puck, and T.A. Fleisher. 2001. Immunophenotypic profiles in families with autoimmune lymphoproliferative syndrome. Blood. 98:2466–2473. 10.1182/blood.v98.8.2466 11588044

[70] Magerus, A., C. Bercher-Brayer, and F. Rieux-Laucat. 2021. The genetic landscape of the FAS pathway deficiencies. Biomed. J. 44:388–399. 10.1016/j.bj.2021.06.005 34171534 PMC8514852

[71] Kuehn, H.S., W. Ouyang, B. Lo, E.K. Deenick, J.E. Niemela, D.T. Avery, J.N. Schickel, D.Q. Tran, J. Stoddard, Y. Zhang, . 2014. Immune dysregulation in human subjects with heterozygous germline mutations in CTLA4. Science. 345:1623–1627. 10.1126/science.1255904 25213377 PMC4371526

[72] Schubert, D., C. Bode, R. Kenefeck, T.Z. Hou, J.B. Wing, A. Kennedy, A. Bulashevska, B.S. Petersen, A.A. Schäffer, B.A. Grüning, . 2014. Autosomal dominant immune dysregulation syndrome in humans with CTLA4 mutations. Nat. Med. 20:1410–1416. 10.1038/nm.3746 25329329 PMC4668597

[73] Schwab, C., A. Gabrysch, P. Olbrich, V. Patiño, K. Warnatz, D. Wolff, A. Hoshino, M. Kobayashi, K. Imai, M. Takagi, . 2018. Phenotype, penetrance, and treatment of 133 cytotoxic T-lymphocyte antigen 4–insufficient subjects. J. Allergy Clin. Immunol. 142:1932–1946. 10.1016/j.jaci.2018.02.055 29729943 PMC6215742

[74] Turnbull, C., J. Bones, M. Stanley, A. Medhavy, H. Wang, A.M.D. Lorenzo, J. Cappello, S. Shanmuganandam, A. Pandey, S. Seneviratne, . 2023. DECTIN-1: A modifier protein in CTLA-4 haploinsufficiency. Sci. Adv. 9:eadi9566. 10.1126/sciadv.adi9566 38055819 PMC10699772

[75] Lopez-Herrera, G., G. Tampella, Q. Pan-Hammarström, P. Herholz, C.M. Trujillo-Vargas, K. Phadwal, A.K. Simon, M. Moutschen, A. Etzioni, A. Mory, . 2012. Deleterious mutations in LRBA are associated with a syndrome of immune deficiency and autoimmunity. Am. J. Hum. Genet. 90:986–1001. 10.1016/j.ajhg.2012.04.015 22608502 PMC3370280

[76] Gámez-Díaz, L., D. August, G. Stepensky, S. Revel-Vilk, M.G. Seidel, M. Noriko, T. Morio, A.J.J. Worth, J. Blessing, F. Van de Veerdonk F, . 2016. The extended phenotype of LPS-responsive beige-like anchor protein (LRBA) deficiency*.* J. Allergy Clin. Immunol. 137:223–230. 10.1016/j.jaci.2015.09.025 26768763

[77] Knox, A.V.C., L.Y. Cominsky, D. Sun, E. Cruz Cabrera, B.E. Nolan, E. Ofray, E. Benetti, C. Visconti, F. Barzaghi, S.D. Rosenzweig, . 2025. One hundred thirty-four germ line PU.1 variants and the agammaglobulinemic patients carrying them. Blood. 145:2549–2560. 10.1182/blood.2024026683 39854693 PMC12163740

[78] Forsyth, K.S., N.E. Toothacre, N. Jiwrajka, A.M. Driscoll, L.A. Shallberg, C. Cunningham-Rundles, S. Barmettler, J. Farmer, J. Verbsky, J. Routes, . 2024. Maintenance of X chromosome inactivation after T cell activation requires NF-κB signaling. Sci. Immunol. 9:eado0398. 10.1126/sciimmunol.ado0398 39365876 PMC12088372

[79] Fieschi, C., S. Dupuis, E. Catherinot, J. Feinberg, J. Bustamante, A. Breiman, F. Altare, R. Baretto, F. Le Deist, S. Kayal, . 2003. Low penetrance, broad resistance, and favorable outcome of interleukin 12 receptor beta1 deficiency: Medical and immunological implications. J. Exp. Med. 197:527–535. 10.1084/jem.20021769 12591909 PMC2193866

[80] de Beaucoudrey, L., A. Samarina, J. Bustamante, A. Cobat, S. Boisson-Dupuis, J. Feinberg, S. Al-Muhsen, L. Jannière, Y. Rose, M. de Suremain, . 2011. Revisiting human IL-12Rbeta1 deficiency: A survey of 141 patients from 30 countries. Medicine. 89:381–402. 10.1097/MD.0b013e3181fdd832 PMC3129625 21057261

[81] Prando, C., A. Samarina, J. Bustamante, S. Boisson-Dupuis, A. Cobat, C. Picard, Z. AlSum, S. Al-Jumaah, S. Al-Hajjar, H. Frayha, . 2013. Inherited IL-12p40 deficiency: Genetic, immunologic, and clinical features of 49 patients from 30 kindreds. Medicine. 92:109–122. 10.1097/MD.0b013e31828a01f9 23429356 PMC3822760

[82] El-Brolosy, M.A., Z. Kontarakis, A. Rossi, C. Kuenne, S. Günther, N. Fukuda, K. Kikhi, G.L.M. Boezio, C.M. Takacs, S.L. Lai, . 2019. Genetic compensation triggered by mutant mRNA degradation. Nature. 568:193–197. 10.1038/s41586-019-1064-z 30944477 PMC6707827

[83] Ma, Z., P. Zhu, H. Shi, L. Guo, Q. Zhang, Y. Chen, S. Chen, Z. Zhang, J. Peng, and J. Chen. 2019. PTC-bearing mRNA elicits a genetic compensation response via Upf3a and COMPASS components. Nature. 568:259–263. 10.1038/s41586-019-1057-y 30944473

[84] Gorman, J.A., C. Hundhausen, M. Kinsman, T. Arkatkar, E.J. Allenspach, C. Clough, S.E. West, K. Thomas, A. Eken, S. Khim, . 2019. The TYK2-P1104A autoimmune protective variant limits coordinate signals required to generate specialized T cell subsets. Front. Immunol. 10:44. 10.3389/fimmu.2019.00044 30740104 PMC6355696

[85] Stewart, O., C. Gruber, H.E. Randolph, R. Patel, M. Ramba, E. Calzoni, L.H. Huang, J. Levy, S. Buta, A. Lee, . 2025. Monoallelic expression can govern penetrance of inborn errors of immunity. Nature. 637:1186–1197. 10.1038/s41586-024-08346-4 39743591 PMC11804961

[86] Rodríguez-Cortez, V.C., L. Del Pino-Molina, J. Rodríguez-Ubreva, L. Ciudad, D. Gómez-Cabrero, C. Company, J.M. Urquiza, J. Tegnér, C. Rodríguez-Gallego, E. López-Granados, . 2015. Monozygotic twins discordant for common variable immunodeficiency reveal impaired DNA demethylation during naïve-to-memory B-cell transition*.* Nat. Commun. 6:7335. 10.1038/ncomms8335 26081581 PMC4557293

[87] Del Pino-Molina, L., J. Rodríguez-Ubreva, J. Torres Canizales, M. Coronel-Díaz, M. Kulis, J.I. Martín-Subero, M. van der Burg, E. Ballestar, E. López-Granados, . 2019. Impaired CpG demethylation in common variable immunodeficiency associates with B cell phenotype and proliferation rate. Front. Immunol. 10:878. 10.3389/fimmu.2019.00878 31105700 PMC6492528

[88] Rodriguez-Ubreva, J., A. Arutyunyan, M.J. Bonder, L. Del Pino-Molina, S.J. Clark, C. de la Calle-Fabregat, L. Garcia-Alonso, L.F. Handfield, L. Ciudad, E. Andrés-León, . 2022. Single-cell Atlas of common variable immunodeficiency shows germinal center-associated epigenetic dysregulation in B-cell responses*.* Nat. Commun. 13:1779. 10.1038/s41467-022-29450-x 35365635 PMC8975885

[89] Fremond, M.L. and N. Nathan. 2021. COPA syndrome, 5 years after: Where are we? Jt. Bone Spine. 88:105070. 10.1016/j.jbspin.2020.09.002 32919065

[90] Fremond, M.L. and Y.J. Crow. 2021. STING-mediated lung inflammation and beyond*.* J. Clin. Immunol. 41:501–514. 10.1007/s10875-021-00974-z 33532887

[91] Simchoni, N., S. Koide, M. Likhite, Y. Kuchitsu, S. Kadirvel, C.S. Law, B.M. Elicker, S. Kurra, M.M.-K. Wong, B. Yuan, . 2025. The common HAQ STING allele prevents clinical penetrance of COPA syndrome. J. Exp. Med. 222:e20242179. 10.1084/jem.20242179 40014299 PMC11867111

[92] Ranjbarnejad, T., H. Abolhassani, R. Sherkat, M. Salehi, F. Ranjbarnejad, N. Vatandoost, and M. Sharifi. 2025. Exploring monogenic, polygenic, and epigenetic models of common variable immunodeficiency. Hum. Mutat. 2025:1725906. 10.1155/humu/1725906 40265101 PMC12014265

[93] Salzer, U., and B. Grimbacher. 2021. TACI deficiency - a complex system out of balance. Curr. Opin. Immunol. 71:81–88. 10.1016/j.coi.2021.06.004 34247095

[94] Bogaert, D.J.A., M. Dullaers, B.N. Lambrecht, K.Y. Vermaelen, E. De Baere, and F. Haerynck. 2016. Genes associated with common variable immunodeficiency: One diagnosis to rule them all? J. Med. Genet. 53:575–590. 10.1136/jmedgenet-2015-103690 27250108

[95] de Valles-Ibáñez, G., A. Esteve-Solé, M. Piquer, E.A. González-Navarro, J. Hernandez-Rodriguez, H. Laayouni, E. González-Roca, A.M. Plaza-Martin, Á. Deyà-Martínez, A. Martín-Nalda, . 2018. Evaluating the genetics of common variable immunodeficiency: Monogenetic model and beyond. Front. Immunol. 9:636. 10.3389/fimmu.2018.00636 29867916 PMC5960686

[96] Nomani, H., Z. Deng, B. Navetta-Modrov, J. Yang, M. Yun, O. Aroniadis, P. Gorevic, I. Aksentijevich, and Q. Yao. 2023. Implications of combined NOD2 and other gene mutations in autoinflammatory diseases. Front. Immunol. 14:1265404. 10.3389/fimmu.2023.1265404 37928541 PMC10620916

[97] Massaad, M.J., J. Zhou, D. Tsuchimoto, J. Chou, H. Jabara, E. Janssen, S. Glauzy, B.G. Olson, H. Morbach, T.K. Ohsumi, . 2016. Deficiency of base excision repair enzyme NEIL3 drives increased predisposition to autoimmunity. J. Clin. Invest. 126:4219–4236. 10.1172/JCI85647 27760045 PMC5096910

[98] Ameratunga, R., W. Koopmans, S.-T. Woon, E. Leung, K. Lehnert, C.A. Slade, J.C. Tempany, A. Enders, R. Steele, P. Browett, . 2017. Epistatic interactions between mutations of TACI (TNFRSF13B) and TCF3 result in a severe primary immunodeficiency disorder and systemic lupus erythematosus. Clin. Transl. Immunol. 6:e159. 10.1038/cti.2017.41 PMC5671988 29114388

[99] Clementi, R., L. Dagna, U. Dianzani, L. Dupré, I. Dianzani, M. Ponzoni, A. Cometa, A. Chiocchetti, M.G. Sabbadini, C. Rugarli, . 2004. Inherited perforin and Fas mutations in a patient with autoimmune lymphoproliferative syndrome and lymphoma. N. Engl. J. Med. 351:1419–1424. 10.1056/NEJMoa041432 15459303

[100] Cerutti, E., M.F. Campagnoli, M. Ferretti, E. Garelli, N. Crescenzio, A. Rosolen, A. Chiocchetti, M.J. Lenardo, U. Ramenghi, and U. Dianzani. 2007. Co-inherited mutations of Fas and caspase-10 in development of the autoimmune lymphoproliferative syndrome. BMC Immunol. 8:28–29. 10.1186/1471-2172-8-28 17999750 PMC2211507

[101] Hoffmann, F., P. Lohse, S. Stojanov, Y.S. Shin, E.D. Renner, A. Kéry, S. Zellerer, and B.H. Belohradsky. 2005. Identification of a novel mevalonate kinase gene mutation in combination with the common MVK V377I substitution and the low-penetrance TNFRSF1A R92Q mutation. Eur. J. Hum. Genet. 13:510–512. 10.1038/sj.ejhg.5201352 15657603

[102] Hoyos-Bachiloglu, R., J. Chou, C.N. Sodroski, A. Beano, W. Bainter, M. Angelova, E. Al Idrissi, M.K. Habazi, H.A. Alghamdi, F. Almanjomi, . 2017. A digenic human immunodeficiency characterized by IFNAR1 and IFNGR2 mutations. J. Clin. Invest. 127:4415–4420. 10.1172/JCI93486 29106381 PMC5707159

[103] Rigaud, S., E. Lopez-Granados, S. Sibéril, G. Gloire, N. Lambert, C. Lenoir, C. Synaeve, M. Stacey, L. Fugger, J.-L. Stephan, . 2011. Human X-linked variable immunodeficiency caused by a hypomorphic mutation in XIAP in association with a rare polymorphism in CD40LG. Blood. 118:252–261. 10.1182/blood-2011-01-328849 21543760

[104] Christodoulou, K., A.E. Wiskin, J. Gibson, W. Tapper, C. Willis, N.A. Afzal, R. Upstill-Goddard, J.W. Holloway, M.A. Simpson, R.M. Beattie, . 2013. Next generation exome sequencing of paediatric inflammatory bowel disease patients identifies rare and novel variants in candidate genes. Gut. 62:977–984. 10.1136/gutjnl-2011-301833 22543157 PMC3686259

[105] Boisson, B., Y.-D. Wang, A. Bosompem, C.S. Ma, A. Lim, T. Kochetkov, S.G. Tangye, J.-L. Casanova, and M.E. Conley. 2013. A recurrent dominant negative E47 mutation causes agammaglobulinemia and BCR- B cells. J. Clin. Invest. 123:4781–4785. 10.1172/JCI71927 24216514 PMC3809807

[106] Revel-Vilk, S., U. Fischer, B. Keller, S. Nabhani, L. Gámez-Díaz, A. Rensing-Ehl, M. Gombert, A. Hönscheid, H. Saleh, A. Shaag, . 2015. Autoimmune lymphoproliferative syndrome-like disease in patients with LRBA mutation. Clin. Immunol. 159:84–92. 10.1016/j.clim.2015.04.007 25931386

[107] Ripen, A.M., M.Y. Chiow, P.R. Rama Rao, and S.B. Mohamad. 2021. Revealing chronic granulomatous disease in a patient with Williams-Beuren syndrome using whole exome sequencing. Front. Immunol. 12:778133. 10.3389/fimmu.2021.778133 34804071 PMC8599285

[108] Yang, Y., D.M. Muzny, F. Xia, Z. Niu, R. Person, Y. Ding, P. Ward, A. Braxton, M. Wang, C. Buhay, . 2014. Molecular findings among patients referred for clinical whole-exome sequencing. JAMA - J. Am. Med. Assoc. 312:1870–1879. 10.1001/jama.2014.14601 PMC4326249 25326635

[109] Posey, J.E., J.A. Rosenfeld, R.A. James, M. Bainbridge, Z. Niu, X. Wang, S. Dhar, W. Wiszniewski, Z.H.C. Akdemir, T. Gambin, . 2016. Molecular diagnostic experience of whole-exome sequencing in adult patients. Genet. Med. 18:678–685. 10.1038/gim.2015.142 26633545 PMC4892996

[110] Chinn, I.K., R.P. Sanders, A. Stray-Pedersen, Z.H. Coban-Akdemir, V.H.D. Kim, H. Dadi, C.M. Roifman, T. Quigg, J.R. Lupski, J.S. Orange, and I.C. Hanson. 2017. Novel combined immune deficiency and radiation sensitivity blended phenotype in an adult with biallelic variations in ZAP70 and RNF168. Front. Immunol. 8:576. 10.3389/fimmu.2017.00576 28603521 PMC5445153

[111] Rae, W., D. Ward, C. Mattocks, R.J. Pengelly, E. Eren, S.V. Patel, S.N. Faust, D. Hunt, and A.P. Williams. 2018. Clinical efficacy of a next-generation sequencing gene panel for primary immunodeficiency diagnostics. Clin. Genet. 93:647–655. 10.1111/cge.13163 29077208

[112] Migita, K., K. Agematsu, J. Masumoto, H. Ida, S. Honda, Y. Jiuchi, Y. Izumi, Y. Maeda, R. Uehara, Y. Nakamura, . 2013. The contribution of SAA1 polymorphisms to familial mediterranean fever susceptibility in the Japanese population. PLoS ONE. 8:e55227. 10.1371/journal.pone.0055227 23437051 PMC3577815

[113] Timberlake, A.T., J. Choi, S. Zaidi, Q. Lu, C. Nelson-Williams, E.D. Brooks, K. Bilguvar, I. Tikhonova, S. Mane, J.F. Yang, . 2016. Two locus inheritance of non-syndromic midline craniosynostosis via rare SMAD6 and common BMP2 alleles. Elife. 5:e20125. 10.7554/eLife.20125 27606499 PMC5045293

[114] Cady, J., P. Allred, T. Bali, A. Pestronk, A. Goate, T.M. Miller, R.D. Mitra, J. Ravits, M.B. Harms, and R.H. Baloh. 2015. Amyotrophic lateral sclerosis onset is influenced by the burden of rare variants in known amyotrophic lateral sclerosis genes. Ann. Neurol. 77:100–113. 10.1002/ana.24306 25382069 PMC4293318

[115] Girard, S.L., P.A. Dion, C.V. Bourassa, S. Geoffroy, P. Lachance-Touchette, A. Barhdadi, M. Langlois, R. Joober, M.-O. Krebs, M.P. Dubé, and G.A. Rouleau. 2015. Mutation burden of rare variants in schizophrenia candidate genes. PLoS One. 10:e0128988. 10.1371/journal.pone.0128988 26039597 PMC4454531

[116] Guo, M.H., L. Plummer, Y.M. Chan, J.N. Hirschhorn, and M.F. Lippincott. 2018. Burden testing of rare variants identified through exome sequencing via publicly available control data. Am. J. Hum. Genet. 103:522–534. 10.1016/j.ajhg.2018.08.016 30269813 PMC6174288

[117] Kreins, A.Y., M.J. Ciancanelli, S. Okada, X.-F. Kong, N. Ramírez-Alejo, S.S. Kilic, J. El Baghdadi, S. Nonoyama, S.A. Mahdaviani, F. Ailal, . 2015. Human TYK2 deficiency: Mycobacterial and viral infections without hyper-IgE syndrome. J. Exp. Med. 212:1641–1662. 10.1084/jem.20140280 26304966 PMC4577846

[118] Boisson-Dupuis, S., N. Ramirez-Alejo, Z. Li, E. Patin, G. Rao, G. Kerner, C.K. Lim, D.N. Krementsov, N. Hernandez, C.S. Ma, . 2018. Tuberculosis and impaired IL-23–dependent IFN-γ immunity in humans homozygous for a common TYK2 missense variant. Sci. Immunol. 3:eaau8714. 10.1126/sciimmunol.aau8714 30578352 PMC6341984

[119] Kerner, G., N. Ramirez-Alejo, Y. Seeleuthner, R. Yang, M. Ogishi, A. Cobat, E. Patin, L. Quintana-Murci, S. Boisson-Dupuis, J.L. Casanova, and L. Abel. 2019. Homozygosity for TYK2 P1104A underlies tuberculosis in about 1% of patients in a cohort of European ancestry. Proc. Natl. Acad. Sci. USA. 116:10430–10434. 10.1073/pnas.1903561116 31068474 PMC6534977

[120] Contreras-Cubas, C., H. García-Ortiz, R. Velázquez-Cruz, F. Barajas-Olmos, P. Baca, A. Martínez-Hernández, R.E. Barbosa-Cobos, J. Ramírez-Bello, M.A. López-Hernández, Y. Svyryd, . 2019. Catalytically impaired TYK2 variants are protective against childhood- and adult-onset systemic lupus erythematosus in Mexicans. Sci. Rep. 9:12165. 10.1038/s41598-019-48451-3 31434951 PMC6704113

[121] Pellenz, F.M., C. Dieter, G.C.K. Duarte, L.H. Canani, B.M. de Souza, and D. Crispim. 2021. The rs2304256 polymorphism in TYK2 gene is associated with protection for type 1 diabetes mellitus. Diabetes Metab. J. 45:899–908. 10.4093/dmj.2020.0194 34225445 PMC8640150

[122] Cheng, Y.H.H., S.C. Bohaczuk, and A.B. Stergachis. 2024. Functional categorization of gene regulatory variants that cause Mendelian conditions. Hum. Genet. 143:559–605. 10.1007/s00439-023-02639-w 38436667 PMC11078748

[123] Thaventhiran, J.E.D., H. Lango Allen, O.S. Burren, W. Rae, D. Greene, E. Staples, Z. Zhang, J.H.R. Farmery, I. Simeoni, E. Rivers, . 2020. Whole-genome sequencing of a sporadic primary immunodeficiency cohort. Nature. 583:90–95. 10.1038/s41586-020-2265-1 32499645 PMC7334047

[124] Telenti, A., and J. di Iulio. 2020. Regulatory genome variants in human susceptibility to infection. Hum. Genet. 139:759–768. 10.1007/s00439-019-02091-9 31807864 PMC7272256

[125] Green, M.R., E. Camilleri, M.K. Gandhi, J. Peake, and L.R. Griffiths. 2011. A novel immunodeficiency disorder characterized by genetic amplification of interleukin 25. Genes Immun. 12:663–666. 10.1038/gene.2011.50 21776014

[126] Orange, J.S., J.T. Glessner, E. Resnick, K.E. Sullivan, M. Lucas, B. Ferry, C.E. Kim, C. Hou, F. Wang, R. Chiavacci, . 2011. Genome-wide association identifies diverse causes of common variable immunodeficiency. J. Allergy Clin. Immunol. 127:1360–1367.e6. 10.1016/j.jaci.2011.02.039 21497890 PMC3646656

[127] Keller, M., J. Glessner, E. Resnick, E. Perez, H. Chapel, M. Lucas, K.E. Sullivan, C. Cunningham-Rundles, J.S. Orange, and H. Hakonarson. 2014. Burden of copy number variation in common variable immunodeficiency. Clin. Exp. Immunol. 177:269–271. 10.1111/cei.12255 24329717 PMC4089176

[128] Al-Mousa, H., M. Abouelhoda, D.M. Monies, N. Al-Tassan, A. Al-Ghonaium, B. Al-Saud, H. Al-Dhekri, R. Arnaout, S. Al-Muhsen, N. Ades, . 2016. Unbiased targeted next-generation sequencing molecular approach for primary immunodeficiency diseases. J. Allergy Clin. Immunol. 137:1780–1787. 10.1016/j.jaci.2015.12.1310 26915675

[129] Bradshaw, G., R.R. Lualhati, C.L. Albury, N. Maksemous, D. Roos-Araujo, R.A. Smith, M.C. Benton, D.A. Eccles, R.A. Lea, H.G. Sutherland, . 2018. Exome sequencing diagnoses X-linked Moesin-associated immunodeficiency in a primary immunodeficiency case. Front. Immunol. 9:420. 10.3389/fimmu.2018.00420 29556235 PMC5845094

[130] Chinen, J., and W.T. Shearer. 2010. Secondary immunodeficiencies, including HIV infection. J. Allergy Clin. Immunol. 125:S195–S203. 10.1016/j.jaci.2009.08.040 20042227 PMC6151868

[131] Zhang, X., D. Bogunovic, B. Payelle-Brogard, V. Francois-Newton, S.D. Speer, C. Yuan, S. Volpi, Z. Li, O. Sanal, D. Mansouri, . 2015. Human intracellular ISG15 prevents interferon-α/β over-amplification and auto-inflammation. Nature. 517:89–93. 10.1038/nature13801 25307056 PMC4303590

[132] Israel, L., Y. Wang, K. Bulek, E. Della Mina, Z. Zhang, V. Pedergnana, M. Chrabieh, N.A. Lemmens, V. Sancho-Shimizu, M. Descatoire, . 2017. Human adaptive immunity rescues an inborn error of innate immunity. Cell. 168:789–800.e10. 10.1016/j.cell.2017.01.039 28235196 PMC5328639

[133] Picard, C., H. von Bernuth, P. Ghandil, M. Chrabieh, O. Levy, P.D. Arkwright, D. McDonald, R.S. Geha, H. Takada, J.C. Krause, . 2010. Clinical features and outcome of patients with IRAK-4 and MyD88 deficiency. Medicine. 89:403–425. 10.1097/MD.0b013e3181fd8ec3 21057262 PMC3103888

[134] Sung, L., S.S. Weitzman, M. Petric, and S.M. King. 2001. The role of infections in primary hemophagocytic lymphohistiocytosis: A case series and review of the literature. Clin. Infect. Dis. 33:1644–1648. 10.1086/323675 11595993

[135] Felgentreff, K., S.N. Baxi, Y.N. Lee, K. Dobbs, L.A. Henderson, K. Csomos, E.N. Tsitsikov, M. Armanios, J.E. Walter, and L.D. Notarangelo. 2016. Ligase-4 deficiency causes distinctive immune abnormalities in asymptomatic individuals. J. Clin. Immunol. 36:341–353. 10.1007/s10875-016-0266-5 27063650 PMC4842108

[136] Bökenkamp, A., M. deJong, J.A.E. van Wijk, D. Block, J.M. van Hagen, and M. Ludwig. 2005. R561C missense mutation in the SMARCAL1 gene associated with mild Schimke immuno-osseous dysplasia. Pediatr. Nephrol. 20:1724–1728. 10.1007/s00467-005-2047-x 16237566

[137] Dekel, B., S. Metsuyanim, N. Goldstein, N. Pode-Shakked, Y. Kovalski, Y. Cohen, M. Davidovits, and Y. Anikster. 2008. Schimke immuno-osseous dysplasia: Expression of SMARCAL1 in blood and kidney provides novel insight into disease phenotype. Pediatr. Res. 63:398–403. 10.1203/PDR.0b013e31816721cc 18356746

[138] Elizonod, L.I., K.S. Cho, W. Zhang, J. Yan, C. Huang, Y. Huang, K. Choi, E.A. Sloan, K. Deguchi, S. Lou, . 2009. Schimke immuno-osseous dysplasia: SMARCAL1 loss-of-function and phenotypic correlation*.* J. Med. Genet. 46:49–59. 10.1136/jmg.2008.060095 18805831

[139] Baradaran-Heravi, A., K.S. Cho, B. Tolhuis, M. Sanyal, O. Morozova, M. Morimoto, L.I. Elizondo, D. Bridgewater, J. Lubieniecka, K. Beirnes, . 2012. Penetrance of biallelic SMARCAL1 mutations is associated with environmental and genetic disturbances of gene expression. Hum. Mol. Genet. 21:2572–2587. 10.1093/hmg/dds083 22378147 PMC3349428

[140] Abel, L., S. Plancoulaine, E. Jouanguy, S.-Y. Zhang, N. Mahfoufi, N. Nicolas, V. Sancho-Shimizu, A. Alcaïs, Y. Guo, A. Cardon, . 2010. Age-dependent mendelian predisposition to herpes simplex virus type 1 encephalitis in childhood. J. Pediatr. 157:623–629.e1. 10.1016/j.jpeds.2010.04.020 20553844

[141] Bradley, H., L.E. Markowitz, T. Gibson, and G.M. McQuillan. 2014. Seroprevalence of herpes simplex virus types 1 and 2-United States, 1999-2010. J. Infect. Dis. 209:325–333. 10.1093/infdis/jit458 24136792

[142] Casrouge, A., S.-Y. Zhang, C. Eidenschenk, E. Jouanguy, A. Puel, K. Yang, A. Alcais, C. Picard, N. Mahfoufi, N. Nicolas, . 2006. Herpes simplex virus encephalitis in human UNC-93B deficiency. Science. 314:308–312. 10.1126/science.1128346 16973841

[143] Zhang, S.-Y., E. Jouanguy, S. Ugolini, A. Smahi, G. Elain, P. Romero, D. Segal, V. Sancho-Shimizu, L. Lorenzo, A. Puel, . 2007. TLR3 deficiency in patients with herpes simplex encephalitis. Science. 317:1522–1527. 10.1126/science.1139522 17872438

[144] Guo, Y., M. Audry, M. Ciancanelli, L. Alsina, J. Azevedo, M. Herman, E. Anguiano, V. Sancho-Shimizu, L. Lorenzo, E. Pauwels, . 2011. Herpes simplex virus encephalitis in a patient with complete TLR3 deficiency: TLR3 is otherwise redundant in protective immunity. J. Exp. Med. 208:2083–2098. 10.1084/jem.20101568 21911422 PMC3182056

[145] Sancho-Shimizu, V., R. Pérez de Diego, L. Lorenzo, R. Halwani, A. Alangari, E. Israelsson, S. Fabrega, A. Cardon, J. Maluenda, M. Tatematsu, . 2011. Herpes simplex encephalitis in children with autosomal recessive and dominant TRIF deficiency. J. Clin. Invest. 121:4889–4902. 10.1172/JCI59259 22105173 PMC3226004

[146] Herman, M., M. Ciancanelli, Y.H. Ou, L. Lorenzo, M. Klaudel-Dreszler, E. Pauwels, V. Sancho-Shimizu, R. Pérez de Diego, A. Abhyankar, E. Israelsson, . 2012. Heterozygous TBK1 mutations impair TLR3 immunity and underlie herpes simplex encephalitis of childhood. J. Exp. Med. 209:1567–1582. 10.1084/jem.20111316 22851595 PMC3428952

[147] Lim, H.K., M. Seppänen, T. Hautala, M.J. Ciancanelli, Y. Itan, F.G. Lafaille, W. Dell, L. Lorenzo, M. Byun, E. Pauwels, . 2014. TLR3 deficiency in herpes simplex encephalitis: High allelic heterogeneity and recurrence risk. Neurology. 83:1888–1897. 10.1212/WNL.0000000000000999 25339207 PMC4248460

[148] Andersen, L.L., N. Mørk, L.S. Reinert, E. Kofod-Olsen, R. Narita, S.E. Jørgensen, K.A. Skipper, K. Höning, H.H. Gad, L. Østergaard, . 2015. Functional IRF3 deficiency in a patient with herpes simplex encephalitis. J. Exp. Med. 212:1371–1379. 10.1084/jem.20142274 26216125 PMC4548062

[149] Mørk, N., E. Kofod-Olsen, K.B. Sørensen, E. Bach, T.F. Ørntoft, L. Østergaard, S.R. Paludan, M. Christiansen, and T.H. Mogensen. 2015. Mutations in the TLR3 signaling pathway and beyond in adult patients with herpes simplex encephalitis. Genes Immun. 16:552–566. 10.1038/gene.2015.46 26513235

[150] Whitley, R.J., and D.W. Kimberlin. 2005. Herpes simplex encephalitis: Children and adolescents. Semin. Pediatr. Infect. Dis. 16:17–23. 10.1053/j.spid.2004.09.007 15685145

[151] Belkaid, Y., and T. Hand. 2015. Role of the microbiota in immunity and inflammation Yasmine. Cell. 157:121–141. 10.1016/j.cell.2014.03.011 PMC4056765 24679531

[152] Gilbert, J.A., M.J. Blaser, J.G. Caporaso, J.K. Jansson, S.V. Lynch, and R. Knight. 2018. Current understanding of the human microbiome. Nat. Med. 24:392–400. 10.1038/nm.4517 29634682 PMC7043356

[153] Jørgensen, S.F., M. Trøseid, M. Kummen, J.A. Anmarkrud, A.E. Michelsen, L.T. Osnes, K. Holm, M.L. Høivik, A. Rashidi, C.P. Dahl, . 2016. Altered gut microbiota profile in common variable immunodeficiency associates with levels of lipopolysaccharide and markers of systemic immune activation. Mucosal Immunol. 9:1455–1465. 10.1038/mi.2016.18 26982597

[154] Fiedorová, K., M. Radvanský, J. Bosák, H. Grombiříková, E. Němcová, P. Králíčková, M. Černochová, I. Kotásková, M. Lexa, J. Litzman, . 2019. Bacterial but not fungal gut microbiota alterations are associated with common variable immunodeficiency (CVID) phenotype*.* Front. Immunol. 10:1914. 10.3389/fimmu.2019.01914 31456808 PMC6700332

[155] Sanchez, D.A., K. Rotella, C. Toribio, M. Hernandez, and C. Cunningham-Rundles. 2023. Characterization of infectious and non-infectious gastrointestinal disease in common variable immunodeficiency: Analysis of 114 patient cohort. Front. Immunol. 14:1209570. 10.3389/fimmu.2023.1209570 37711607 PMC10498782

[156] Roser, M., H. Ritchie, and B. Dadonaite. 2019. Child & Infant Mortality. Our World In Data.

[157] Mensa-Vilaró, A., M. Bravo García-Morato, O. de la Calle-Martin, C. Franco-Jarava, M.T. Martínez-Saavedra, L.I. González-Granado, E. González-Roca, J.L. Fuster, L. Alsina, O.M. Mutchinick, . 2019. Unexpected relevant role of gene mosaicism in patients with primary immunodeficiency diseases. J. Allergy Clin. Immunol. 143:359–368. 10.1016/j.jaci.2018.09.009 30273710

[158] Cooper, M.A. 2025. Somatic mosaicism in genetic errors of immunity. J. Allergy Clin. Immunol. 155:759–767. 10.1016/j.jaci.2024.11.038 39724970 PMC12020649

[159] Torreggiani, S., F.S. Castellan, I. Aksentijevich, and D.B. Beck. 2024. Somatic mutations in autoinflammatory and autoimmune disease. Nat. Rev. Rheumatol. 20:683–698. 10.1038/s41584-024-01168-8 39394526

[160] Uberti, J., W.D. Peterson Jr, J.J. Lightbody, and R.M. Johnson. 1983. A phenotypically normal revertant of an adenosine deaminase-deficient lymphoblast cell line. J. Immunol. 130:2866–2870. 6854019

[161] Arredondo-Vega, F.X., J. Kurtzberg, S. Chaffee, I. Santisteban, E. Reisner, M.S. Povey, and M.S. Hershfield. 1990. Paradoxical expression of adenosine deaminase in T cells cultured from a patient with adenosine deaminase deficiency and combine immunodeficiency. J. Clin. Invest. 86:444–452. 10.1172/JCI114730 1974554 PMC296746

[162] Hirschhorn, R., D.R. Yang, A. Israni, M.L. Huie, and D.R. Ownby. 1994. Somatic mosaicism for a newly identified splice-site mutation in a patient with adenosine deaminase-deficient immunodeficiency and spontaneous clinical recovery. Am. J. Hum. Genet. 55:59–68. 8023852 PMC1918232

[163] Puck, J.M., A.E. Pepper, P.M. Bédard, and R. Laframboise. 1995. Female germ line mosaicism as the origin of a unique IL-2 receptor gamma-chain mutation causing X-linked severe combined immunodeficiency. J. Clin. Invest. 95:895–899. 10.1172/JCI117740 7860773 PMC295580

[164] O’Marcaigh, A.S., J.M. Puck, A.E. Pepper, K. De Santes, and M.J. Cowan. 1997. Maternal mosaicism for a novel interleukin-2 receptor gamma-chain mutation causing X-linked severe combined immunodeficiency in a Navajo kindred. J. Clin. Immunol. 17:29–33. 10.1023/a:1027332327827 9049783

[165] de Koning, H.D., M.E. van Gijn, M. Stoffels, J. Jongekrijg, PLJM Zeeuwen, M.G. Elferink, I.J. Nijman, P.A.M. Jansen, K. Neveling, J.W.M. van der Meer, . 2015. Myeloid lineage–restricted somatic mosaicism of NLRP3 mutations in patients with variant Schnitzler syndrome. J. Allergy Clin. Immunol. 135:561–564. 10.1016/j.jaci.2014.07.050 25239704

[166] Rowczenio, D.M., S.M. Gomes, J.I. Aróstegui, A. Mensa-Vilaro, E. Omoyinmi, H. Trojer, A. Baginska, A. Baroja-Mazo, P. Pelegrin, S. Savic, . 2017. Late-onset cryopyrin-associated periodic syndromes caused by somatic NLRP3 mosaicism-UK single center experience. Front. Immunol. 8:1410. 10.3389/fimmu.2017.01410 29163488 PMC5671490

[167] Hsu, A.P., K.J. Sowerwine, M.G. Lawrence, J. Davis, C.J. Henderson, K.A. Zarember, M. Garofalo, J.I. Gallin, D.B. Kuhns, T. Heller, . 2013. Intermediate phenotypes in patients with autosomal dominant hyper-IgE syndrome caused by somatic mosaicism. J. Allergy Clin. Immunol. 131:1586–1593. 10.1016/j.jaci.2013.02.038 23623265 PMC4103905

[168] Walker, S., C. Wang, T. Walradt, B.S. Hong, J.R. Tanner, J.L. Levinsohn, G. Goh, A. Subtil, S.R. Lessin, W.R. Heymann, . 2016. Identification of a gain-of-function STAT3 mutation (p.Y640F) in lymphocytic variant hypereosinophilic syndrome. Blood. 127:948–951. 10.1182/blood-2015-06-654277 26702067 PMC4760095

[169] Holzelova, E., C. Vonarbourg, M.-C. Stolzenberg, P.D. Arkwright, F. Selz, A.M. Prieur, S. Blanche, J. Bartunkova, E. Vilmer, A. Fischer, . 2004. Autoimmune lymphoproliferative syndrome with somatic Fas mutations. N. Engl. J. Med. 351:1409–1418. 10.1056/NEJMoa040036 15459302

[170] Dowdell, K.C., J.E. Niemela, S. Price, J. Davis, R.L. Hornung, J.B. Oliveira, J.M. Puck, E.S. Jaffe, S. Pittaluga, J.I. Cohen, . 2010. Somatic FAS mutations are common in patients with genetically undefined autoimmune lymphoproliferative syndrome. Blood. 115:5164–5169. 10.1182/blood-2010-01-263145 20360470 PMC2892951

[171] Wolach, B., Y. Scharf, R. Gavrieli, M. de Boer, and D. Roos. 2005. Unusual late presentation of X-linked chronic granulomatous disease in an adult female with a somatic mosaic for a novel mutation in CYBB. Blood. 105:61–66. 10.1182/blood-2004-02-0675 15308575

[172] Kadowaki, T., H. Ohnishi, N. Kawamoto, T. Hori, K. Nishimura, C. Kobayashi, T. Shigemura, S. Ogata, Y. Inoue, T. Kawai, . 2018. Haploinsufficiency of A20 causes autoinflammatory and autoimmune disorders. J. Allergy Clin. Immunol. 141:1485–1488.e11. 10.1016/j.jaci.2017.10.039 29241730

[173] Kontzias, A., S.K. Zarabi, C. Calabrese, Y. Wang, L. Judis, Q. Yao, and Y.W. Cheng. 2019. Somatic mosaicism in adult-onset TNF receptor-associated periodic syndrome (TRAPS). Mol. Genet. Genomic Med. 7:e791. 10.1002/mgg3.791 31397119 PMC6687656

[174] Materna-Kiryluk, A., A. Pollak, K. Gawalski, A. Szczawinska-Poplonyk, Z. Rydzynska, A. Sosnowska, B. Cukrowska, P. Gasperowicz, E. Konopka, B. Pietrucha, . 2021. Mosaic IL6ST variant inducing constitutive GP130 cytokine receptor signaling as a cause of neonatal onset immunodeficiency with autoinflammation and dysmorphy. Hum. Mol. Genet. 30:226–233. 10.1093/hmg/ddab035 33517393

[175] Aluri, J., A. Bach, S. Kaviany, L. Chiquetto Paracatu, M. Kitcharoensakkul, M.A. Walkiewicz, C.D. Putnam, M. Shinawi, N. Saucier, E.M. Rizzi, . 2021. Immunodeficiency and bone marrow failure with mosaic and germline TLR8 gain of function. Blood. 137:2450–2462. 10.1182/blood.2020009620 33512449 PMC8109013

[176] Benavides-Nieto, M., F. Adam, E. Martin, C. Boussard, C. Lagresle-Peyrou, I. Callebaut, A. Kauskot, C. Repérant, M. Feng, J.-C. Bordet, . 2024. Somatic RAP1B gain-of-function variant underlies isolated thrombocytopenia and immunodeficiency. J. Clin. Invest. 134:e169994. 10.1172/JCI169994 39225097 PMC11364392

[177] Beck, D.B., M.A. Ferrada, K.A. Sikora, A.K. Ombrello, J.C. Collins, W. Pei, N. Balanda, D.L. Ross, D. Ospina Cardona, Z. Wu, . 2020. Somatic mutations in UBA1 and severe adult-onset autoinflammatory disease. N. Engl. J. Med. 383:2628–2638. 10.1056/NEJMoa2026834 33108101 PMC7847551

[178] Koster, M.J., M.J. Samec, and K.J. Warrington. 2023. VEXAS syndrome-A review of pathophysiology, presentation, and prognosis. J. Clin. Rheumatol. 29:298–306. 10.1097/RHU.0000000000001905 36251488

[179] Magerus-Chatinet, A., B. Neven, M.-C. Stolzenberg, C. Daussy, P.D. Arkwright, N. Lanzarotti, C. Schaffner, S. Cluet-Dennetiere, F. Haerynck, G. Michel, . 2011. Onset of autoimmune lymphoproliferative syndrome (ALPS) in humans as a consequence of genetic defect accumulation. J. Clin. Invest. 121:106–112. 10.1172/JCI43752 21183795 PMC3007148

[180] Neven, B., A. Magerus-Chatinet, B. Florkin, D. Gobert, O. Lambotte, L. De Somer, N. Lanzarotti, M.-C. Stolzenberg, B. Bader-Meunier, N. Aladjidi, . 2011. A survey of 90 patients with autoimmune lymphoproliferative syndrome related to TNFRSF6 mutation. Blood. 118:4798–4807. 10.1182/blood-2011-04-347641 21885602

[181] Hauck, F., A. Magerus-Chatinet, S. Vicca, A. Rensing-Ehl, A. Roesen-Wolff, J. Roesler, and F. Rieux-Laucat. 2013. Somatic loss of heterozygosity, but not haploinsufficiency alone, leads to full-blown autoimmune lymphoproliferative syndrome in 1 of 12 family members with FAS start codon mutation. Clin. Immunol. 147:61–68. 10.1016/j.clim.2013.02.019 23524443

[182] Martínez-Feito, A., J. Melero, S. Mora-Díaz, C. Rodríguez-Vigil, R. Elduayen, L.I. González-Granado, D. Pérez-Méndez, E. Sánchez-Zapardiel, R. Ruiz-García, M. Menchén, . 2016. Autoimmune lymphoproliferative syndrome due to somatic FAS mutation (ALPS-sFAS) combined with a germline caspase-10 (CASP10) variation. Immunobiology. 221:40–47. 10.1016/j.imbio.2015.08.004 26323380

[183] Jing, H., Q. Zhang, Y. Zhang, B.J. Hill, C.G. Dove, E.W. Gelfand, T.P. Atkinson, G. Uzel, H.F. Matthews, P.J. Mustillo, . 2014. Somatic reversion in dedicator of cytokinesis 8 immunodeficiency modulates disease phenotype. J. Allergy Clin. Immunol. 133:1667–1675. 10.1016/j.jaci.2014.03.025 24797421 PMC4132167

[184] Pillay, B.A., M. Fusaro, P.E. Gray, A.L. Statham, L. Burnett, L. Bezrodnik, A. Kane, W. Tong, C. Abdo, S. Winter, . 2021. Somatic reversion of pathogenic DOCK8 variants alters lymphocyte differentiation and function to effectively cure DOCK8 deficiency. J. Clin. Invest. 131:e142434. 10.1172/JCI142434 33290277 PMC7843233

[185] Ariga, T., N. Oda, K. Yamaguchi, N. Kawamura, H. Kikuta, S. Taniuchi, Y. Kobayashi, K. Terada, H. Ikeda, M.S. Hershfield, . 2001. T-cell lines from 2 patients with adenosine deaminase (ADA) deficiency showed the restoration of ADA activity resulted from the reversion of an inherited mutation. Blood. 97:2896–2899. 10.1182/blood.v97.9.2896 11313286

[186] Stephan, V., V. Wahn, F. Le Deist, U. Dirksen, B. Broker, I. Müller-Fleckenstein, G. Horneff, H. Schroten, A. Fischer, and G. de Saint Basile. 1996. Atypical X-linked severe combined immunodeficiency due to possible spontaneous reversion of the genetic defect in T cells. N. Engl. J. Med. 335:1563–1567. 10.1056/NEJM199611213352104 8900089

[187] Speckmann, C., U. Pannicke, E. Wiech, K. Schwarz, P. Fisch, W. Friedrich, T. Niehues, K. Gilmour, K. Buiting, M. Schlesier, . 2008. Clinical and immunologic consequences of a somatic reversion in a patient with X-linked severe combined immunodeficiency. Blood. 112:4090–4097. 10.1182/blood-2008-04-153361 18728247

[188] Ariga, T., T. Kondoh, K. Yamaguchi, M. Yamada, S. Sasaki, D.L. Nelson, H. Ikeda, K. Kobayashi, H. Moriuchi, and Y. Sakiyama. 2001. Spontaneous in vivo reversion of an inherited mutation in the Wiskott-Aldrich syndrome. J. Immunol. 166:5245–5249. 10.4049/jimmunol.166.8.5245 11290809

[189] Boztug, K., U. Baumann, M. Ballmaier, D. Webster, I. Sandrock, R. Jacobs, T. Lion, S. Preuner, M. Germeshausen, G. Hansen, . 2007. Large granular lymphocyte proliferation and revertant mosaicism: Two rare events in a Wiskott-Aldrich syndrome patient. Haematologica. 92:43–45. 10.3324/haematol.11222 17405757

[190] Tone, Y., T. Wada, F. Shibata, T. Toma, Y. Hashida, Y. Kasahara, S. Koizumi, and A. Yachie. 2007. Somatic revertant mosaicism in a patient with leukocyte adhesion deficiency type 1. Blood. 109:1182–1184. 10.1182/blood-2007-08-039057 17244687

[191] Nishikomori, R., H. Akutagawa, K. Maruyama, M. Nakata-Hizume, K. Ohmori, K. Mizuno, A. Yachie, T. Yasumi, T. Kusunoki, T. Heike, and T. Nakahata. 2004. X-linked ectodermal dysplasia and immunodeficiency caused by reversion mosaicism of NEMO reveals a critical role for NEMO in human T-cell development and/or survival. Blood. 103:4565–4572. 10.1182/blood-2003-10-3655 14726382

[192] Fuchs, S., A. Rensing-Ehl, U. Pannicke, M.R. Lorenz, P. Fisch, Y. Jeelall, J. Rohr, C. Speckmann, T. Vraetz, S. Farmand, . 2015. Omenn syndrome associated with a functional reversion due to a somatic second-site mutation in CARD11 deficiency. Blood. 126:1658–1669. 10.1182/blood-2015-03-631374 26289640 PMC4654427

[193] Catto, L.F.B., G. Borges, A.L. Pinto, D.V. Clé, F. Chahud, B.A. Santana, F.S. Donaires, and R.T. Calado. 2020. Somatic genetic rescue in hematopoietic cells in GATA2 deficiency. Blood. 136:1002–1005. 10.1182/blood.2020005538 32556109

[194] Boztug, K., M. Germeshausen, I. Avedillo Díez, V. Gulacsy, J. Diestelhorst, M. Ballmaier, K. Welte, L. Maródi, L. Chernyshova, and C. Klein. 2008. Multiple independent second-site mutations in two siblings with somatic mosaicism for Wiskott-Aldrich syndrome. Clin. Genet. 74:68–74. 10.1111/j.1399-0004.2008.01019.x 18479478

[195] Miyazawa, H., and T. Wada. 2021. Reversion mosaicism in primary immunodeficiency diseases. Front. Immunol. 12:783022. 10.3389/fimmu.2021.783022 34868061 PMC8635092

[196] Ban, S.A., E. Salzer, M.M. Eibl, A. Linder, C.B. Geier, E. Santos-Valente, W. Garncarz, T. Lion, R. Ott, C. Seelbach, . 2014. Combined immunodeficiency evolving into predominant CD4+ lymphopenia caused by somatic chimerism in JAK3. J. Clin. Immunol. 34:941–953. 10.1007/s10875-014-0088-2 25205547 PMC4220108

[197] McDermott, D.H., J.-L. Gao, Q. Liu, M. Siwicki, C. Martens, P. Jacobs, D. Velez, E. Yim, C.R. Bryke, N. Hsu, . 2015. Chromothriptic cure of WHIM syndrome. Cell. 160:686–699. 10.1016/j.cell.2015.01.014 25662009 PMC4329071

[198] Del Bel, K.L., R.J. Ragotte, A. Saferali, S. Lee, S.M. Vercauteren, S.A. Mostafavi, R.A. Schreiber, J.S. Prendiville, M.S. Phang, J. Halparin, . 2017. JAK1 gain-of-function causes an autosomal dominant immune dysregulatory and hypereosinophilic syndrome. J. Allergy Clin. Immunol. 139:2016–2020 e5. 10.1016/j.jaci.2016.12.957 28111307

[199] Horesh, M.E., M. Martin-Fernandez, C. Gruber, S. Buta, T. Le Voyer, E. Puzenat, H. Lesmana, Y. Wu, A. Richardson, D. Stein, . 2024. Individuals with JAK1 variants are affected by syndromic features encompassing autoimmunity, atopy, colitis, and dermatitis. J. Exp. Med. 221:e20232387. 10.1084/jem.20232387 38563820 PMC10986756

[200] Niemela, J.E., L. Lu, T.A. Fleisher, J. Davis, I. Caminha, M. Natter, L.A. Beer, K.C. Dowdell, S. Pittaluga, M. Raffeld, . 2011. Somatic KRAS mutations associated with a human nonmalignant syndrome of autoimmunity and abnormal leukocyte homeostasis. Blood. 117:2883–2886. 10.1182/blood-2010-07-295501 21079152 PMC3062298

[201] Takagi, M., K. Shinoda, J. Piao, N. Mitsuiki, M. Takagi, K. Matsuda, H. Muramatsu, S. Doisaki, M. Nagasawa, T. Morio, . 2011. Autoimmune lymphoproliferative syndrome-like disease with somatic KRAS mutation. Blood. 117:2887–2890. 10.1182/blood-2010-08-301515 21063026

[202] Shiota, M., X. Yang, M. Kubokawa, T. Morishima, K. Tanaka, M. Mikami, K. Yoshida, M. Kikuchi, K. Izawa, R. Nishikomori, . 2015. Somatic mosaicism for a NRAS mutation associates with disparate clinical features in RAS-associated Leukoproliferative disease: A report of two cases. J. Clin. Immunol. 35:454–458. 10.1007/s10875-015-0163-3 25896945

[203] Ma, C.A., L. Xi, B. Cauff, A. DeZure, A.F. Freeman, S. Hambleton, G. Kleiner, T.R. Leahy, M. O’Sullivan, M. Makiya, . 2017. Somatic STAT5b gain-of-function mutations in early onset nonclonal eosinophilia, urticaria, dermatitis, and diarrhea. Blood. 129:650–653. 10.1182/blood-2016-09-737817 27956386 PMC5290989

[204] Gimelbrant, A., J.N. Hutchinson, B.R. Thompson, and A. Chess. 2007. Widespread monoallelic expression on human autosomes. Science. 318:1136–1140. 10.1126/science.1148910 18006746

[205] Reinius, B., and R. Sandberg. 2015. Random monoallelic expression of autosomal genes: Stochastic transcription and allele-level regulation. Nat. Rev. Genet. 16:653–664. 10.1038/nrg3888 26442639

[206] Deng, Q., D. Ramsköld, B. Reinius, and R. Sandberg. 2014. Single-cell RNA-seq reveals dynamic, random monoallelic gene expression in mammalian cells. Science. 343:193–196. 10.1126/science.1245316 24408435

[207] Jeffries, A.R., L.W. Perfect, J. Ledderose, L.C. Schalkwyk, N.J. Bray, J. Mill, and J. Price. 2012. Stochastic choice of allelic expression in human neural stem cells. Stem Cells. 30:1938–1947. 10.1002/stem.1155 22714879

[208] Borel, C., P.G. Ferreira, F. Santoni, O. Delaneau, A. Fort, K.Y. Popadin, M. Garieri, E. Falconnet, P. Ribaux, M. Guipponi, . 2015. Biased allelic expression in human primary fibroblast single cells. Am. J. Hum. Genet. 96:70–80. 10.1016/j.ajhg.2014.12.001 25557783 PMC4289680

[209] Reinius, B., J.E. Mold, D. Ramsköld, Q. Deng, P. Johnsson, J. Michaëlsson, J. Frisén, and R. Sandberg. 2016. Analysis of allelic expression patterns in clonal somatic cells by single-cell RNA-seq. Nat. Genet. 48:1430–1435. 10.1038/ng.3678 27668657 PMC5117254

[210] Gruber, C.N., J.J.A. Calis, S. Buta, G. Evrony, J.C. Martin, S.A. Uhl, R. Caron, L. Jarchin, D. Dunkin, R. Phelps, . 2020. Complex autoinflammatory syndrome unveils fundamental principles of JAK1 kinase transcriptional and biochemical function. Immunity. 53:672–684.e11. 10.1016/j.immuni.2020.07.006 32750333 PMC7398039

[211] Nag, A., V. Savova, H.L. Fung, A. Miron, G.C. Yuan, K. Zhang, and A.A. Gimelbrant. 2013. Chromatin signature of widespread monoallelic expression. Elife. 2:e01256. 10.7554/eLife.01256 24381246 PMC3873816

[212] Gendrel, A.-V., M. Attia, C.-J. Chen, P. Diabangouaya, N. Servant, E. Barillot, and E. Heard. 2014. Developmental dynamics and disease potential of random monoallelic gene expression. Dev. Cell. 28:366–380. 10.1016/j.devcel.2014.01.016 24576422

[213] Savova, V., S. Vinogradova, D. Pruss, A.A. Gimelbrant, and L.A. Weiss. 2017. Risk alleles of genes with monoallelic expression are enriched in gain-of-function variants and depleted in loss-of-function variants for neurodevelopmental disorders. Mol. Psychiatry. 22:1785–1794. 10.1038/mp.2017.13 28265118 PMC5589474

[214] Adegbola, A.A., G.F. Cox, E.M. Bradshaw, D.A. Hafler, A. Gimelbrant, and A. Chess. 2015. Monoallelic expression of the human FOXP2 speech gene. Proc. Natl. Acad. Sci. USA. 112:6848–6854. 10.1073/pnas.1411270111 25422445 PMC4460484

